# The phytopathogen *Xanthomonas campestris* senses and effluxes salicylic acid via a sensor HepR and an RND family efflux pump to promote virulence in host plants

**DOI:** 10.1002/mlf2.12140

**Published:** 2024-09-16

**Authors:** Kai Song, Ruifang Li, Ying Cui, Bo Chen, Lian Zhou, Wenying Han, Bo‐Le Jiang, Ya‐Wen He

**Affiliations:** ^1^ State Key Laboratory of Microbial Metabolism, Joint International Research Laboratory of Metabolic and Developmental Sciences, School of Life Sciences and Biotechnology Shanghai Jiao Tong University Shanghai China; ^2^ Guangxi Key Laboratory of Biology for Crop Diseases and Insect Pests, Plant Protection Research Institute Guangxi Academy of Agricultural Sciences Nanning China; ^3^ Zhiyuan Innovative Research Center Shanghai Jiao Tong University Shanghai China; ^4^ State Key Laboratory for Conservation and Utilization of Subtropical Agro‐bioresources, College of Life Science and Technology Guangxi University Nanning China

**Keywords:** efflux, quorum sensing, salicylic acid, sensor, *Xanthomonas campestris*

## Abstract

Salicylic acid (SA) plays an essential role in plant defense against biotrophic and semi‐biotrophic pathogens. Following pathogen recognition, SA biosynthesis dramatically increases at the infection site of the host plant. The manner in which pathogens sense and tolerate the onslaught of SA stress to survive in the plant following infection remains to be understood. The objective of this work was to determine how the model phytopathogen *Xanthomonas campestris* pv. *campestris* (Xcc) senses and effluxes SA during infection inside host plants. First, RNA‐Seq analysis identified an SA‐responsive operon Xcc4167–Xcc4171, encoding a MarR family transcription factor HepR and an RND (resistance‐nodulation‐cell division) family efflux pump HepABCD in Xcc. Electrophoretic mobility shift assays and DNase I footprint analysis revealed that HepR negatively regulated *hepABCD* expression by specifically binding to an AT‐rich region of the promoter of the *hepRABCD* operon, P_hep_. Second, isothermal titration calorimetry and further genetic analysis suggest that HepR is a novel SA sensor. SA binding released HepR from its cognate promoter P_hep_ and then induced the expression of *hepABCD*. Third, the RND family efflux pump HepABCD was responsible for SA efflux. The *hepRABCD* cluster was also involved in the regulation of culture pH and quorum sensing signal diffusible signaling factor turnover. Finally, the *hepRABCD* cluster was transcribed during the XC1 infection of Chinese radish and was required for the full virulence of Xcc in Chinese radish and cabbage. These findings suggest that the ability of Xcc to co‐opt the plant defense signal SA to activate the multidrug efflux pump may have evolved to ensure Xcc survival and virulence in susceptible host plants.

## Impact statement

Salicylic acid (SA) is an essential plant defense hormone. *Xanthomonas campestris* pv. *campestris* (Xcc) is the causal agent of *Brassica* black rot. Xcc infection induces SA biosynthesis in the host plant and SA can directly act on the invading Xcc to disturb its virulence. This study shows that Xcc senses the SA signal via the novel sensor HepR and then activates the efflux pump HepABCD to tolerate the onslaught of SA stress and induce quorum sensing signal turnover. These findings reveal a new strategy to ensure phytopathogen survival and virulence in susceptible host plants.

## INTRODUCTION

Salicylic acid (SA) is a beta‐hydroxy phenolic acid phytohormone that plays an essential role in plant defense against biotrophic and semi‐biotrophic pathogens^1^. Following pathogen recognition, SA biosynthesis is positively regulated, and the local concentrations of SA at the infection site of cucumber and tobacco may reach 150–300 μM in leaf tissues, 300 μM in the roots, and 600 μM in phloem exudates^2^, ^3^. Rice leaf tissue accumulates high levels of free SA, which is not significantly induced by pathogen invasion^4^. The increased concentration of cytosolic SA is then perceived by SA receptors and SA binding proteins in plants^5^, ^6^. Consequently, a wide range of immune responses are activated to protect the host plants from pathogen infection^7^.

Despite SA‐induced immune responses in plants, increasing evidence suggests that the host defense signal SA can directly influence the gene expression and metabolic profiles of invading phytopathogens. SA has been shown to interfere with the transcription of the *repABC* operon, *vir* regulon, and genes associated with quorum sensing (QS) and quorum quenching in *Agrobacterium tumefaciens*, thereby playing a role in attenuating crown gall disease^8^, ^9^. It has also been shown to inhibit biofilm formation, motility, and N‐acyl homoserine lactone‐dependent QS machinery in the phytopathogens *Pectobacterium carotovorum* and *Pectobacterium aroidearum* ^10^. Recently, Song et al.^11^ showed that the exogenous addition of SA or endogenous production of SA in the phytopathogen *Xanthomonas campestris* pv. *campestris* (Xcc) induced RpfB‐dependent QS signal diffusible signaling factor (DSF) turnover and increased the culture pH. SA treatment of Xcc also significantly increased its virulence in cabbage^11^. Although our understanding of the roles of SA in phytopathogens has become increasingly clear, we have less knowledge on how phytopathogens sense SA signals to survive in plants following infection.

Xcc is a Gram‐negative bacterium that causes Brassica black rot, the most dreaded disease of the crucifers worldwide^12^, ^13^. The symptoms include V‐shaped yellow lesions starting from the leaf margins and blackening of the veins. Due to its agricultural importance and comprehensive understanding of virulence^13^, ^14^, ^15^, Xcc was selected as one of the top 10 plant pathogenic bacteria in molecular plant pathology^16^ and a powerful model for research in disease control^13^.

Xcc enters plants via leaf margin hydathodes, stomata, and wounds^17^, ^18^. Once inside the plant, Xcc disperses and colonizes the host vascular system via a DSF‐dependent QS mechanism^14^, ^19^, ^20^. Our previous study showed that Xcc infection induced dramatic SA biosynthesis around the infection site in cabbage. Xcc is not able to degrade SA in vitro^11^. Thus, a high level of SA is one of the stressors encountered during Xcc infection inside host plants. How the phytopathogen Xcc tolerates the onslaught of SA stress to survive in plants remains unclear.

The present study aimed to investigate how Xcc senses and effluxes SA during infection inside host plants. In this study, we identified an SA‐responsive gene cluster in Xcc via RNA‐Seq analysis. The results further showed that this cluster encodes an SA sensor protein and an RND (resistance‐nodulation‐cell division) family efflux pump. Xcc senses and effluxes the host plant SA signal via this gene cluster and induces the QS signal, DSF turnover, to promote virulence in host plants.

## RESULTS

### RNA‐Seq analysis identifies an SA‐responsive gene cluster Xcc4167–Xcc4171

**Figure 1:**
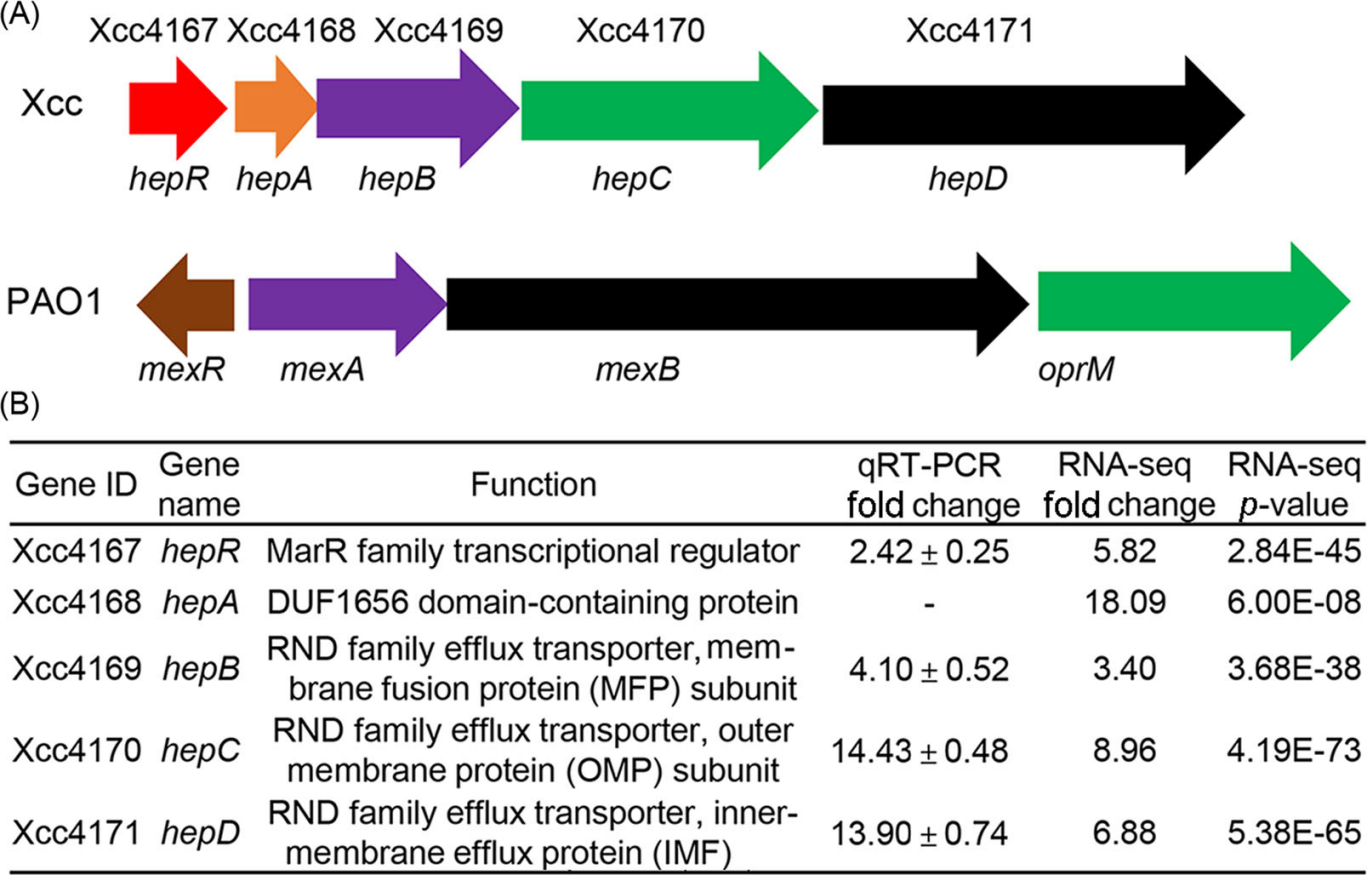
Salicylic acid (SA)‐responsive gene cluster *hepRABCD*. (A) The *hep* gene cluster in *Xanthomonas campestris* pv. *campestris* (Xcc) and its homologous gene cluster in *Pseudomonas aeruginosa* strain PAO1. (B) Fold changes in the *hep* expression level between SA‐treated XC1 and XC1 by RNA‐seq and real‐time quantitative reverse transcriptional PCR (qRT‐PCR) analysis. The Xcc wild‐type strain XC1 was cultured in 50 ml of liquid XYS medium supplemented with 0 or 100 μM SA for 24 h at 28°C.

Previous results showed that SA could induce RpfB‐dependent DSF turnover in Xcc strain XC1^11^. To determine the SA‐regulated genes, total RNA was extracted from XC1 cells grown in XYS medium supplemented with 100 μM SA at 24 hours post inoculation (hpi) and submitted for RNA‐Seq analysis (Figure S1). A total of 203 genes were upregulated by SA, while 231 genes were downregulated by SA (Figure S1). Among them, genes Xcc4167 to Xcc4171 were upregulated 5.82–18.09‐fold by SA (Figure 1A,B). The SA‐induced upregulation of these genes was further verified by real‐time quantitative reverse transcriptional PCR (Figure 1B).

The genes *Xcc4167*–*Xcc4171* encode a MarR family transcription factor, a DUF1656 domain‐containing protein, a membrane fusion protein (MFP) subunit, an outer membrane protein (OMP) subunit, and an inner membrane efflux protein (IMF) subunit of an RND family efflux transporter (Figures 1A and S2). Based on genomic and domain organization analysis, this locus is structurally homologous to the *mexRAB‐oprM* locus, which encodes a type of RND family efflux pump (Figures 1A and S2) and is involved in the efflux of a range of metabolites in *Pseudomonas aeruginosa* PAO1^21^, ^22^. In this study, the genes Xcc4167–Xcc4171 were renamed *hepRABCD* for the hydroxybenzoic acid efflux pump, respectively.

### *hepR* and *hepABCD* are transcribed as a single operon

**Figure 2:**
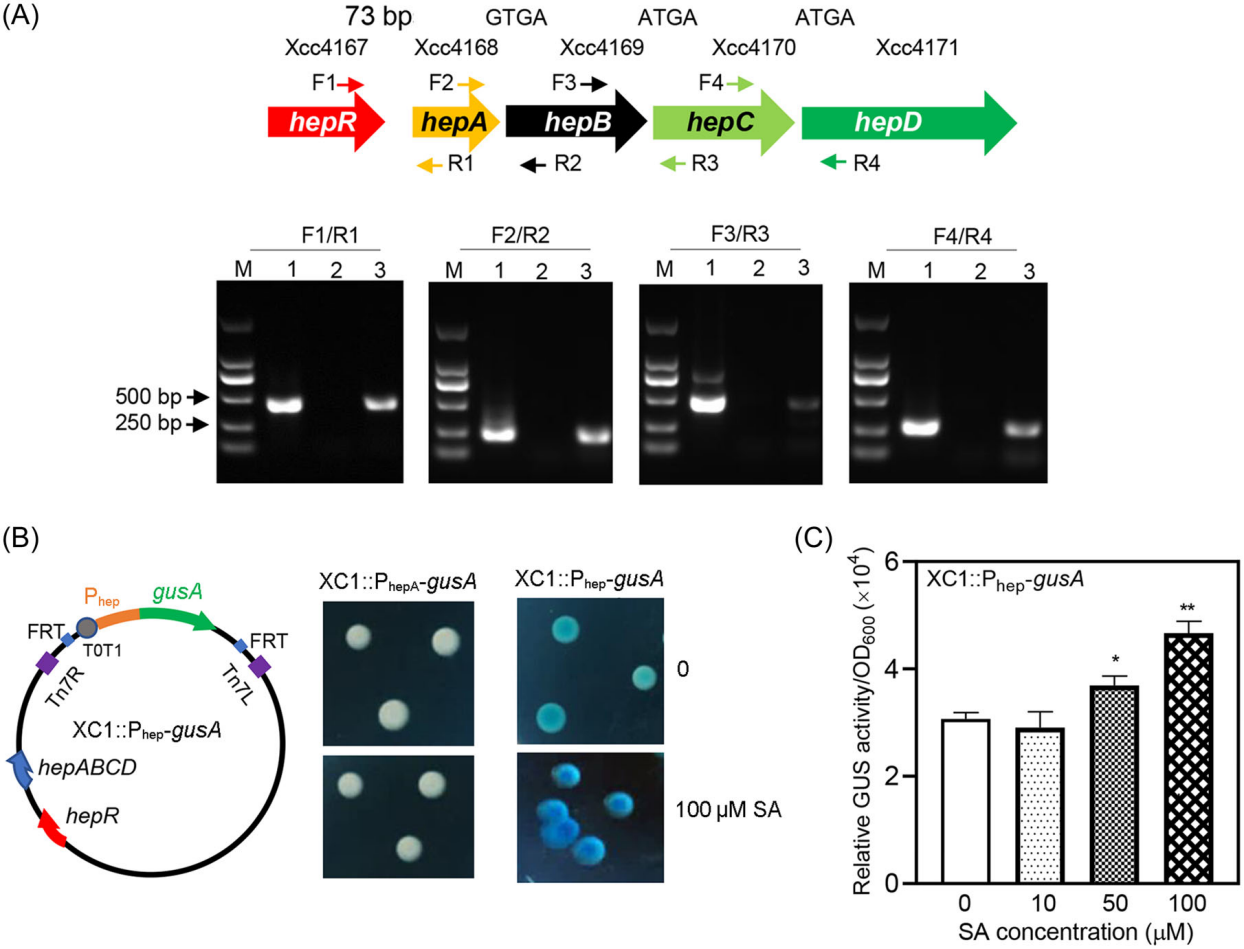
Operon analysis of the *hepRABCD* cluster in Xcc. (A) Co‐transcription of *hepR* and *hepABCD*. Lane 1: PCR products amplified using XC1 genomic DNA as a template. Lane 2: PCR products amplified using the total RNAs of XC1 as a template. Lane 3: RT‐PCR products amplified using the total RNAs of XC1 as a template. (B) Left: A schematic showing the constructed reporter strain to monitor the promoter activity of *hepRABCD*. Right: Colony color in the absence or presence of 100 μM SA. (C) GUS activity of reporter strain XC1::P_hep_‐*gusA* in the presence of 10–100 μM SA. Three independent experiments were conducted. Data are expressed as the mean ± standard deviation of three independent assays. Statistically significant differences are indicated by one (*p* < 0.05) or two asterisks (*p* < 0.01). GUS, β‐glucuronidase.

*hepR and hepABCD* were transcribed in the same orientation in the Xcc genome (Figure 2A). *hepA*, *hepB*, *hepC*, and *hepD* were separated by four base pairs (bp), including GTGA, ATGA, and ATGA (Figure 2A), and *hepR* was separated from *hepA* by 73 bp (Figure 2A). These results suggest that *hepRABCD* is transcribed as a single operon. To verify this hypothesis, reverse transcription PCR (RT‐PCR) was performed using the total RNA extracted from XC1 treated with 100 μM SA, and four DNA fragments corresponding to the four gap regions between *hepR* and *hepD* were identified (Figure 2A).

Furthermore, the promoter‐*gusA* fusion reporter strains, XC1::P_hep_‐*gusA* and XC1::P_hepA_‐*gusA*, were generated to monitor the putative promoter activities of the 531 bp region upstream of *hepR* and the 437 bp region upstream of *hepA*, respectively (Figure 2B). In the absence of SA, basal *gusA*‐dependent β‐glucuronidase (GUS) activity was detected in the reporter strain XC1::P_hep_‐*gusA* but not in reporter strain XC1::P_hepA_‐*gusA* (Figure 2B). In the presence of 100 μM SA, a high level of *gusA*‐dependent GUS activity was detected in strain XC1::P_hep_‐*gusA*, while no GUS activity was detected in strain XC1::P_hepA_‐*gusA* (Figure 2B). These results verified the promoter activity of P_hep_.

Furthermore, using the reporter strain XC1::P_hep_‐*gusA*, our results demonstrated that the transcriptional activities of P_hep_ increased with an SA concentration from 10 to 100 μM (Figure 2C). Taken together, these results suggest that *hepR* and *hepABCD* are transcribed as a single operon from the shared promoter P_hep_.

### HepR negatively regulates *hepABCD* expression by binding specifically to an AT‐rich region of P_hep_

**Figure 3:**
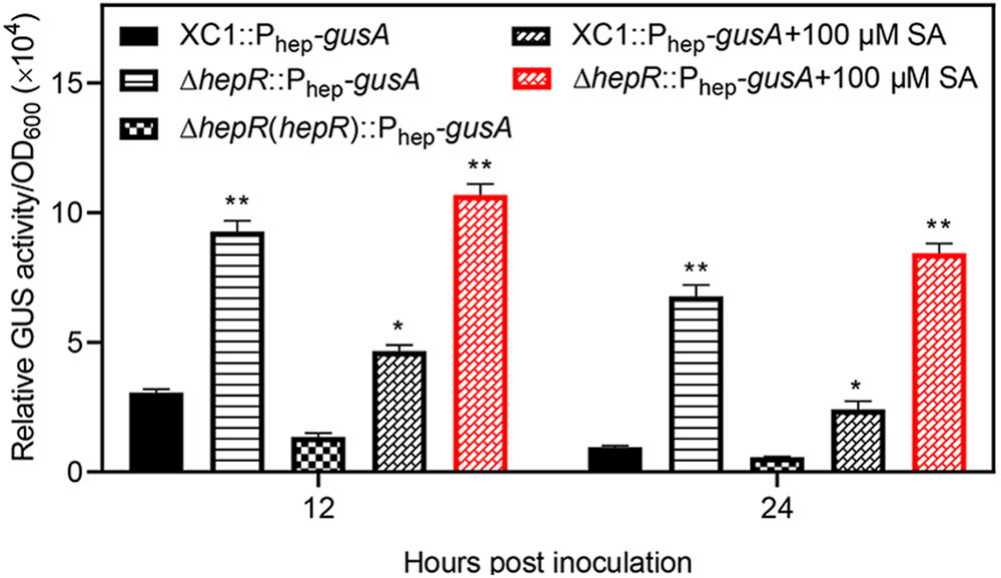
HepR negatively regulates *hepABCD* expression. All strains were grown in an XYS liquid medium in the absence or presence of 100 μM SA. Samples were collected at 12 and 24 hpi, respectively. Data are expressed as the mean ± standard deviation of three independent assays. Statistically significant differences are indicated by one asterisk (*p* < 0.05) or two asterisks (*p* < 0.01). hpi, hours post innoculation.

HepR belongs to the MarR family transcriptional factor. To investigate its role in the expression of *hepABCD*, *hepR* was deleted in the reporter strain XC1::P_hep_‐*gusA* to generate Δ*hepR*::P_hep_‐*gusA*. As shown in Figure 3, the expression level of *hepABCD* indicated by GUS activity in the strain Δ*hepR*::P_hep_‐*gusA* was 3.0–6.9 times higher than that in the strain XC1::P_hep_‐*gusA* at 12‐24 hpi in the absence of SA, suggesting that HepR negatively regulates *hepABCD* expression.

**Figure 4:**
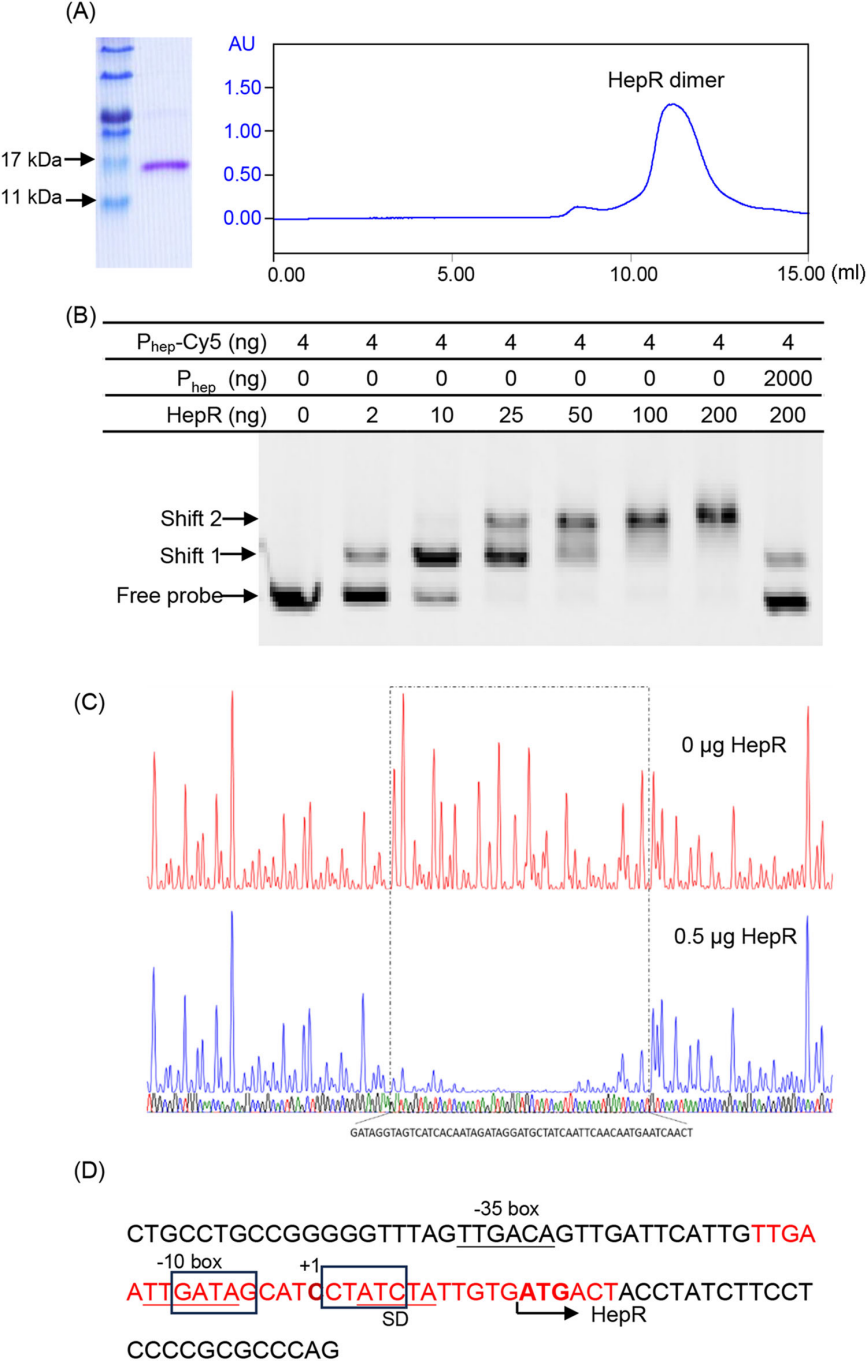
HepR specifically binds to the promoter P_hep_ to negatively regulate *hepRABCD* transcription. (A) Purified HepR dimers. (B) Electrophoretic mobility shift assay (EMSA) using Cy5‐labeled DNA probes P_hep_ (291 bp) with 2–200 ng HepR protein. As a cold probe, 2000 ng P_hep_ was added. (C) DNase I footprint analysis using the 0.5 μg HepR protein and 291‐bp DNA probe P_hep_. (D) HepR binding site in the promoter of *hepRABCD*, P_hep_. The −10/−35 sequences are underlined and labeled with −10 and −35, respectively. The sequence of the protection region is shown in red, and the typical palindromic sequence is indicated by a rectangular box. The transcription start site (+1) was identified using 5′‐RACE. SD, Shine−Dalgarno sequence.

To further investigate how HepR negatively regulates *hepABCD* expression, HepR was expressed via the pET‐28a vector, and His‐tagged HepR was purified using Ni‐NTA resins (Figure 4A). Fast protein liquid chromatography (FPLC) assays revealed that the HepR proteins formed dimers (Figure 4A). Electrophoretic mobility shift assay (EMSA) was then conducted to examine the DNA‐binding activity of HepR to a 291‐bp DNA fragment encompassing the *hep* promoter region, P_hep_ (4 ng). As shown in Figure 4B, a significant shift in labeled DNA was observed in the presence of increasing concentrations (2–200 ng) of HepR. In the presence of high levels of unlabeled probes (2000 ng), no more shifts were observed (Figure 4B). These results indicate specific binding between HepR and P_hep_. To identify the precise HepR binding site, DNase I footprinting analysis was performed. A representative sequencing result is shown in Figure 4C. HepR protected a core 54‐bp DNA region “AGTTGATTCATTGTTGAATTGATAGCATCCTATCTATTGTGATGACTACCTATC” from DNase I digestion.

To determine how HepR binding affected the transcription of the *hep* cluster, 5′‐RACE analyses were first conducted, and the transcription site at C (+1) was identified (Figure 4D). Based on these results, the −10 and −35 boxes within the *hepR* promoter were identified using the program Prokaryote Promoter Prediction (Figure 4D). The identified HepR binding site fully overlapped the −10 and −35 boxes in the promoter P_hep_ (Figure 4D).

**Figure 5:**
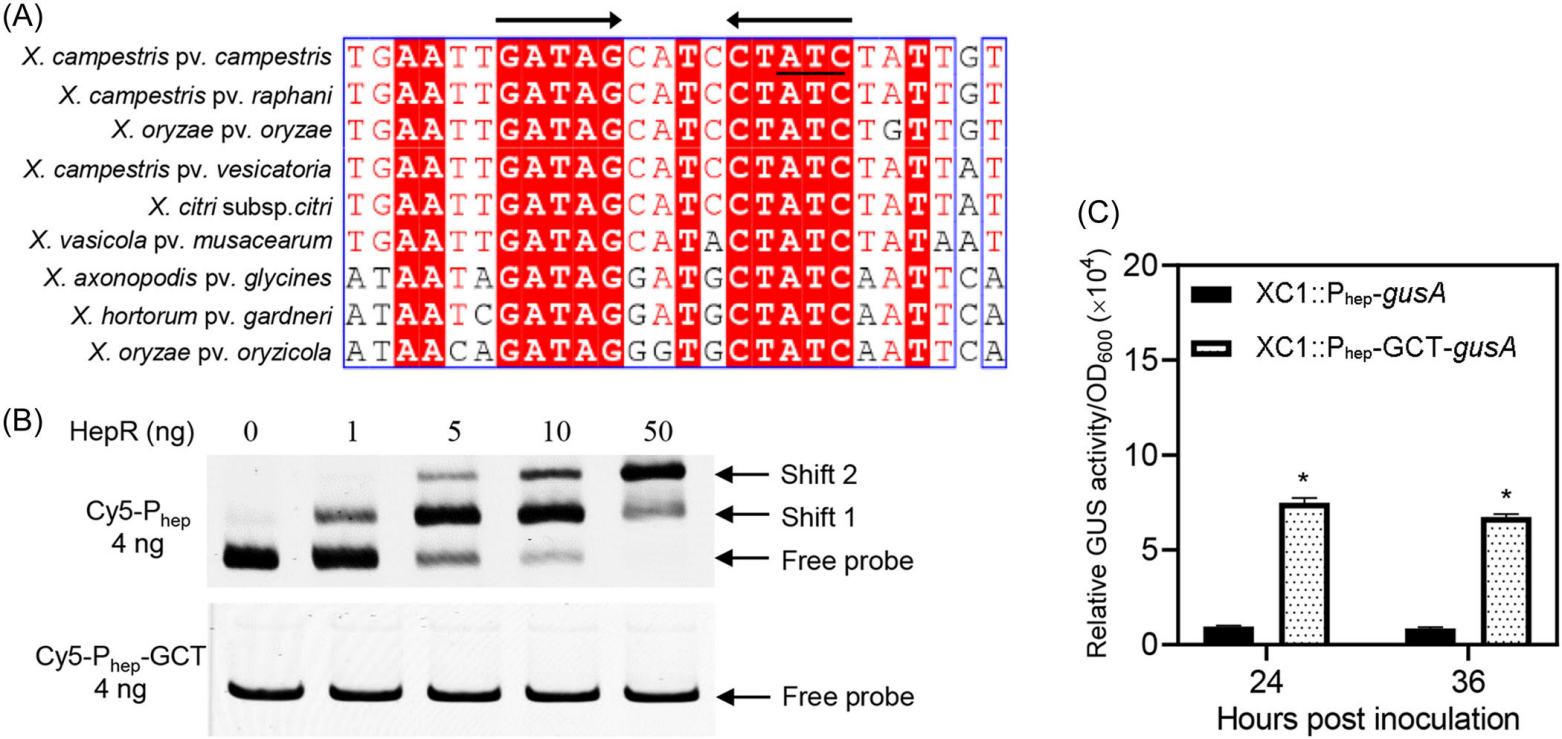
Verification of the HepR‐binding site. (A) The typical palindromic sequence within the 31‐bp binding site. (B) EMSA of the binding between HepR and the mutated Cy5‐labeled probe. The mutated Cy5‐labeled probe is a DNA fragment that converts ATC in the inverted repeat sequence to GCT. (C) The relative β‐glucuronidase activities of the two reporter strains, XC1::P_hep_‐*gusA* and XC1::P_hep_‐GCT‐*gusA*. The averages for three technical repeats with the standard deviation are shown. Statistically significant differences are indicated by one asterisk (*p* < 0.05).

An alignment of the *hep* promoter sequences from nine *Xanthomonas* species revealed a putative inverted repeat sequence of GATAG‐CTATC (Figure 5A). When ATC within the inverted repeat sequence was converted to GCT, the resultant variant was unable to bind to HepR (Figure 5B). Point mutation of the ATC into GCT within the promoter region led to a significantly higher GUS level in reporter strain XC1::P_hep_‐*gusA* (Figure 5C). These results verify the key role of the inverted repeat sequence in HepR binding.

### HepR is a novel SA sensor

**Figure 6:**
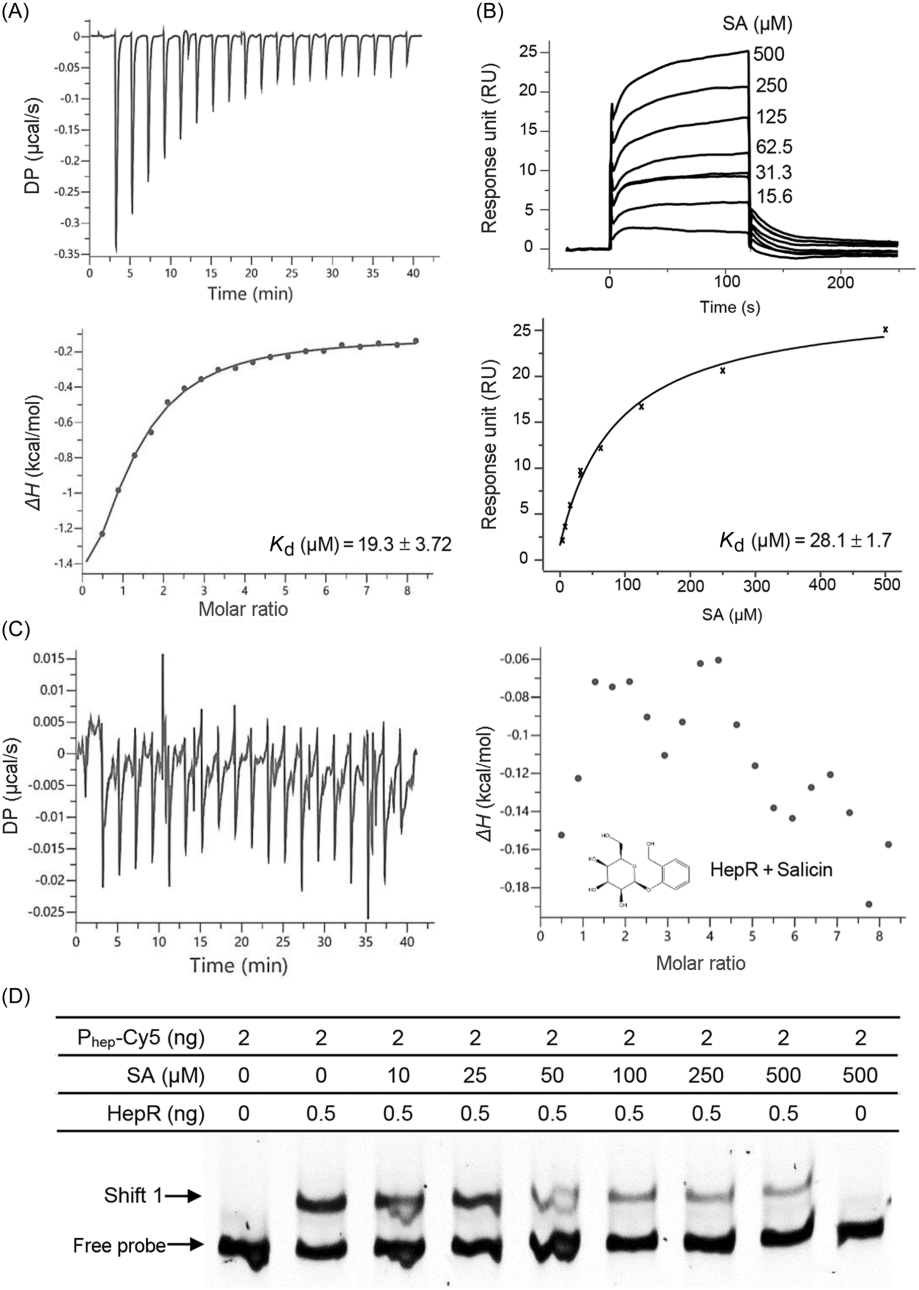
HepR is an SA sensor protein. (A) Isothermal titration calorimetry (ITC) analysis of the binding of SA to HepR. (B) Surface plasmon resonance (SPR) analysis of the binding of SA to HepR. (C) ITC analysis showing no binding between salicin, the SA analog, and HepR. (D) EMSA showing that SA interferes with HepR binding to promoter P_hep_. DP: the measured power difference between the reference cell and the sample cell. Δ*H* represents the heat released at the injection.

HepR is a 147‐amino acid protein belonging to the transcriptional regulator of the MarR family (Figures S2 and S3A). Both isothermal titration calorimetry (ITC) and surface plasmon resonance (SPR) analyses showed that SA could bind HepR dimers, with *K*_d_ values of 19.3 ± 3.72 μM and 28.1 ± 1.7 μM, respectively (Figure 6A,B). In contrast, the SA analog salicin had no binding activity with HepR (Figure 6C). The addition of SA to strain Δ*hepR* had no significant effect on the expression level of *hepABCD* (Figure 3). Furthermore, the addition of SA to the EMSA reaction mixture inhibited binding between HepR and P_hep_ (Figure 6D). These results suggest that HepR is an SA sensor in Xcc.

Previously, SlyA was shown to be an SA sensor in *Salmonella* ^23^, ^24^. SlyA and its homologs in *Escherichia coli*, *Shigella flexneri*, and *Pectobacterium carotovorum* shared conserved amino acids and domain organization (Figure S3B). Although both HepR and SlyA shared similar MarR domain organizations, alignment analysis identified limited identical amino acids (Figure S3B). Furthermore, among SlyA and its homologs, two residues, W^34^ and T^66^, have been shown to be essential for the SA binding of SlyA^24^. However, these two residues were not present in HepR (Figure S3B). Furthermore, genomic analysis showed that the *slyA*‐flanking genes in *Salmonella*, *E*. *coli*, *S*. *flexneri*, and *P*. *carotovorum* were highly conserved. In contrast, the *hepR*‐flanking genes in *Xanthomonas* were completely different from those of *slyA* (Figure S3C). These results suggest that HepR represents a novel SA sensor.

### *hepABCD* is responsible for SA efflux

**Figure 7:**
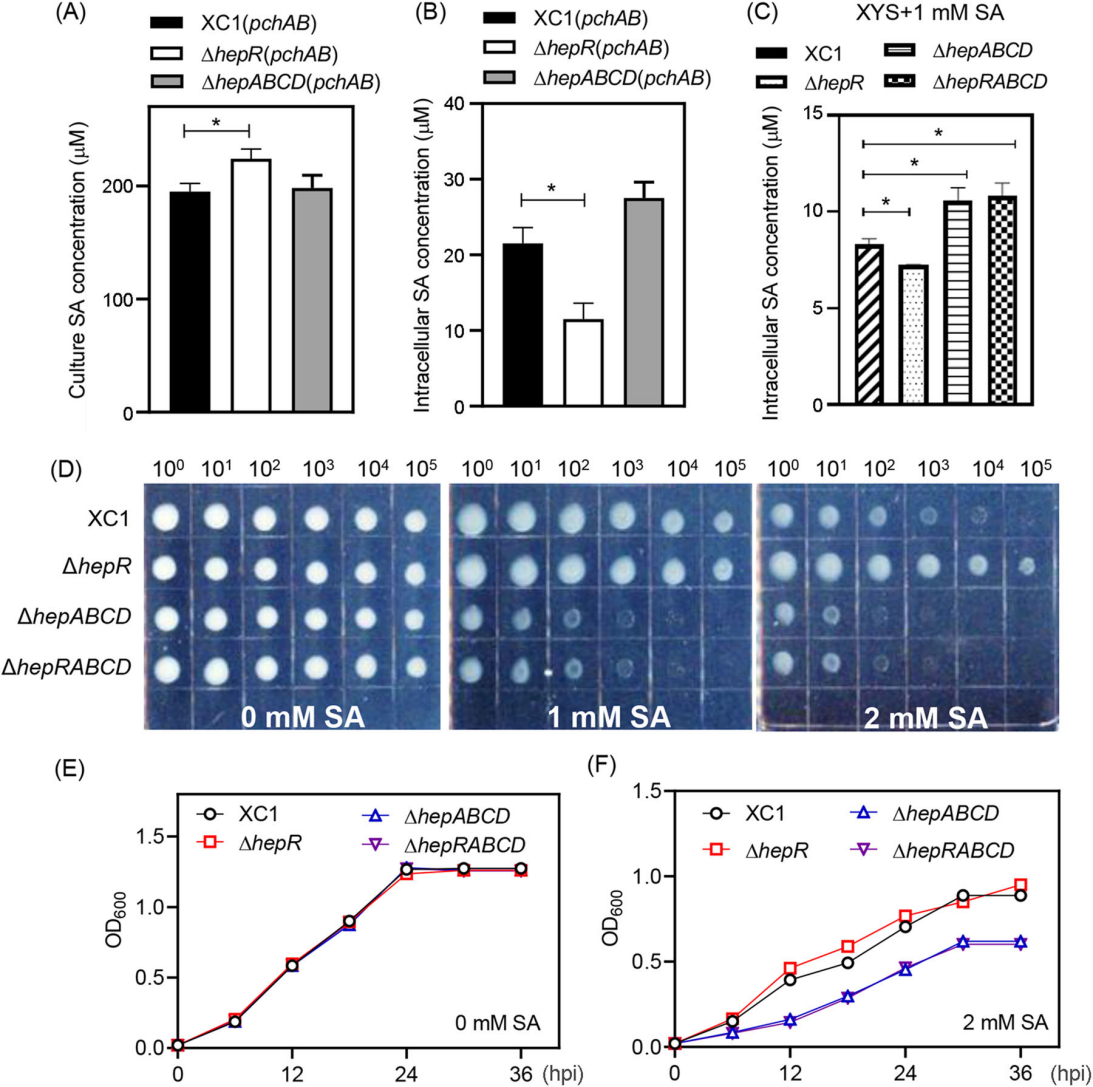
*hepABCD* encodes an SA efflux pump. (A, B) The culture (A) and intracellular (B) SA level of the Xcc strains of XC1(*pchAB*), Δ*hepR*(*pchAB*), and Δ*hepABCD*(*pchAB*). (C) Intracellular SA levels of XC1, Δ*hepR*, Δ*hepABCD*, and Δ*hepRABCD* in the presence of 1 mM SA. (D) Growth of XC1, Δ*hepR*, Δ*hepABCD*, and Δ*hepRABCD* on an XYS agar plate supplemented with 0–2 mM SA. (E, F) Growth of XC1, Δ*hepR*, Δ*hepABCD*, and Δ*hepRABCD* in liquid XYS medium in the absence of SA (E) and in the presence of 2 mM SA (F). The averages for three technical repeats with the standard deviation are shown. Statistically significant differences are indicated by one asterisk (*p* < 0.05).

To further investigate whether *hepABCD* is responsible for SA efflux, the gene cluster *pchAB* from *P*. *aeruginosa* was overexpressed via the plasmid pBBR1MCS‐2 in strains XC1, Δ*hepR*, and Δ*hepABCD* to endogenously produce SA as previously described by Song et al.^11^ The resultant strains XC1 (*pchAB*), Δ*hepR* (*pchAB*), and Δ*hepABCD* (*pchAB*) were examined for the cultural SA level and intracellular SA level at 24 hpi in XYS culture. Our results showed that the level of SA in the cultures of strains XC1 (*pchAB*), Δ*hepR* (*pchAB*), and Δ*hepABCD* (*pchAB*) was 195, 237, and 198 μM, respectively, at 24 hpi in XYS medium (Figure 7A). The intracellular SA levels in the three strains were 21.5, 11.5, and 27.5 μM, respectively (Figure 7B). Furthermore, the intracellular SA levels of XC1, Δ*hepR*, Δ*hepABCD*, and Δ*hepRABCD* were detected in XYS medium supplemented with 1 mM SA. The intracellular SA levels of Δ*hepR*, Δ*hepABCD*, and Δ*hepRABCD* represented 87.3%, 127.1%, and 130.1% of that in XC1, respectively, at 24 hpi (Figure 7C). These results support that *hepABCD* is responsible for SA efflux.

To further verify that *hepABCD* is responsible for SA efflux, the tolerance of Xcc strains to high levels of SA was examined. In the absence of SA, strains XC1, Δ*hepR*, Δ*hepABCD*, and Δ*hepRABCD* displayed similar growth rates on an XYS agar plate (Figure 7D). However, these strains displayed differential growth rates on the XYS agar plate supplemented with 1–2 mM SA. Strain Δ*hepR*, in which the genes *hepABCD* were overexpressed, displayed the highest tolerance to SA among the tested strains. Strains Δ*hepABCD* and Δ*hepRABCD* displayed a significantly lower tolerance to SA than the wild‐type strain XC1 (Figure 7D). Similarly, in the presence of 2 mM SA, wild‐type strain XC1 displayed a significantly higher tolerance than strains Δ*hepABCD* and Δ*hepRABCD* but a significantly lower tolerance than strain Δ*hepR* (Figure 7E,F) in liquid XYS medium. Taken together, these results further support that the gene cluster *hepABCD* is responsible for SA efflux.

### The *hepRABCD* cluster is involved in the regulation of culture pH and DSF turnover

**Figure 8:**
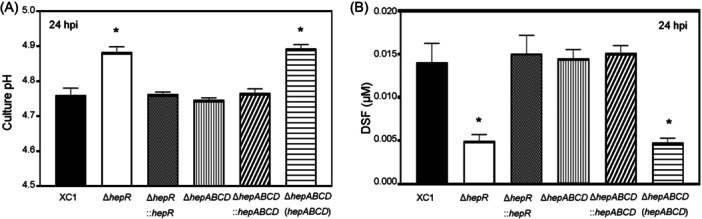
The *hepRABCD* cluster is involved in the regulation of culture pH and diffusible signaling factor (DSF) levels. (A) Culture pH of the Xcc strains in XYS medium at 24 hpi. (B) The DSF level of the Xcc strains in the XYS culture at 24 hpi. The averages for three technical repeats with the standard deviation are shown. Statistically significant differences are indicated by one asterisk (*p* < 0.05).

Our previous results showed that the addition of SA increased the XYS culture pH and induced DSF turnover in Xcc^11^. In this study, to determine whether the *hepRABCD* cluster is involved in the regulation of culture pH and DSF turnover, the culture pH and DSF levels of the XC1‐derived mutant strains, Δ*hepABCD*, Δ*hepR*, and their respective complementation strains, were determined. The deletion of *hepR* (Δ*hepR*) in strain XC1 significantly increased the culture pH at 24 hpi. This was complemented by integrating a single copy of *hepR* (Figure 8A). Deletion of *hepABCD* (Δ*hepABCD*) had no significant effect on culture pH; however, the overexpression of *hepABCD* via the plasmid pBBR1MCS‐2 in the mutant strain Δ*hepABCD* (Δ*hepABCD* (*hepABCD*)) significantly increased the culture pH (Figure 8A). Similarly, deletion of *hepR* (Δ*hepR*) in XC1 significantly decreased the DSF level. This was complemented by integrating a single copy of *hepR* (Figure 8B). Deletion of *hepABCD* (Δ*hepABCD*) in XC1 had no significant effect on the DSF level; however, the overexpression of *hepABCD* in mutant strain Δ*hepABCD* (Δ*hepABCD* (*hepABCD*)) significantly decreased the DSF level (Figure 8B).

The *rpf* cluster is responsible for DSF synthesis, perception, and degradation^14^. In this study, to explore whether deletion of *hepRABCD* affects the expression of *rpf* cluster, a series of *gusA*‐dependent reporter strains were constructed in the strains XC1, Δ*hepR*, and Δ*hepABCD*, respectively (Figure S4). GUS analysis revealed no significant difference among these strains (Figure S4). These results suggest that the *rpf* transcription is not regulated by *hepRABCD*.

### The *hepRABCD* cluster is transcribed during XC1 infection of Chinese radish

**Figure 9:**
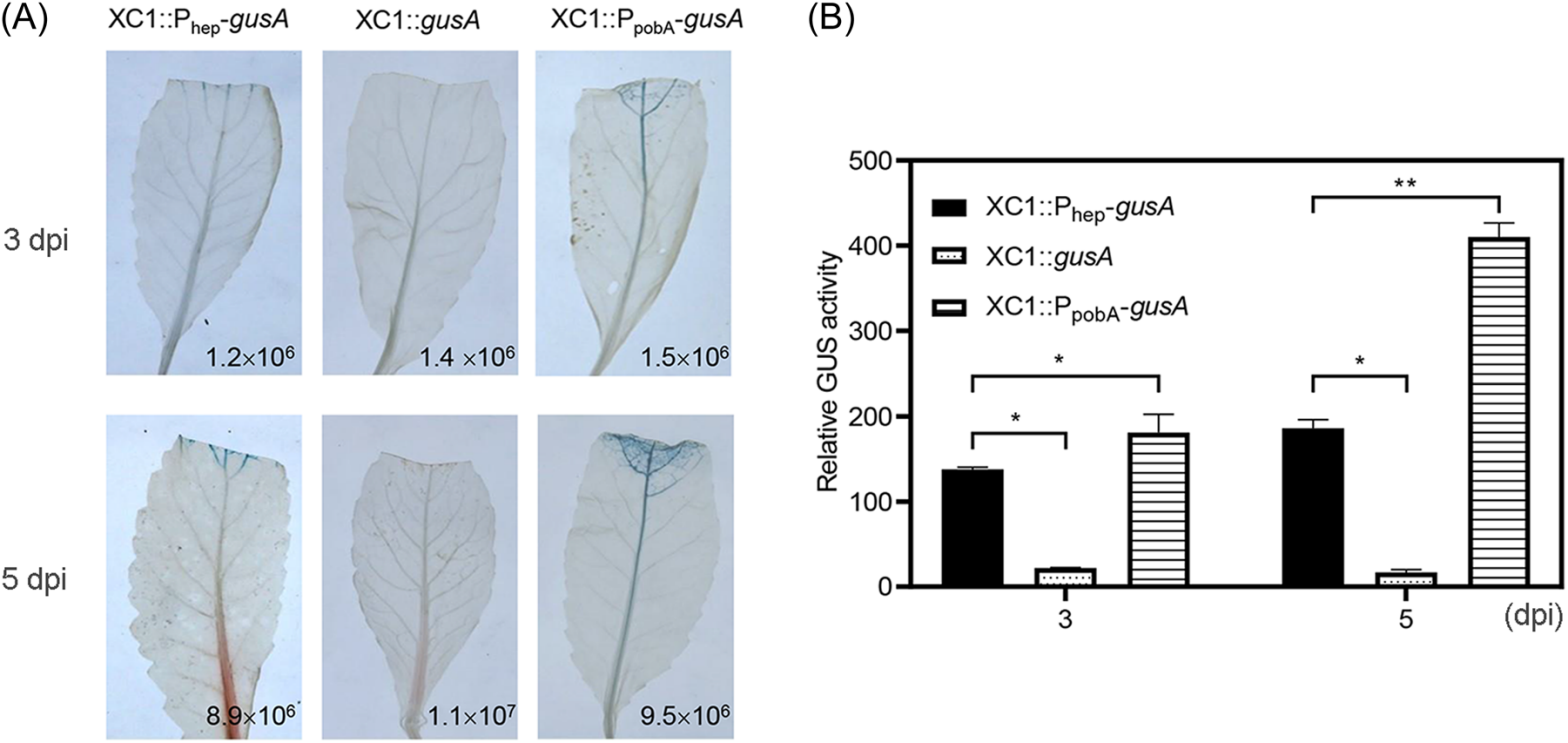
*hepRABCD* is transcribed during XC1 infection inside Chinese radish. (A) β‐Glucuronidase (GUS) histochemical staining in infected Chinese radish leaves at 3 and 5 dpi. The numbers in the lower right corner indicate the bacterial colony formation units (CFUs) in the infected leaf tissues. (B) Quantitative analysis of different promoter‐driven GUS activities per 10^8^ CFUs of the Xcc strains inside Chinese radish using 4‐methylumbelliferyl‐β‐d‐glucuronide as a substrate at 3 and 5 dpi. XC1::*gusA* represents a negative control strain containing a nonpromoter‐driven *gusA* gene. XC1::P_pobA_‐*gusA* represents a positive control strain containing a *gusA* gene driven by the promoter of *pobA* in Xcc. Statistically significant differences are indicated by one asterisk (*p* < 0.05) or two asterisks (*p* < 0.01).

Xcc is a xylem‐dwelling phytopathogen. To elucidate the role of the *hepRABCD* cluster in Xcc pathogenesis, the expression of this cluster was determined during disease development. To this end, strain XC1::P_hep_‐*gusA* was introduced into Chinese radish. XC1::*gusA* carrying a non‐promoter‐driven *gusA* gene and the previously constructed *pobA* reporter strain XC1::P_pobA_‐*gusA* were used as the negative and positive control strains, respectively^25^. P_hep_‐dependent GUS expression was detected in the infected leaf tissues using histochemical staining analysis (Figure 9A). Subsequent GUS quantitative assays demonstrated that the expression level of the *hep* cluster was lower than that of *pobA* inside infected tissues (Figure 9B).

### The *hepRABCD* cluster is required for the full virulence of XC1 in cabbage and Chinese radish

**Figure 10:**
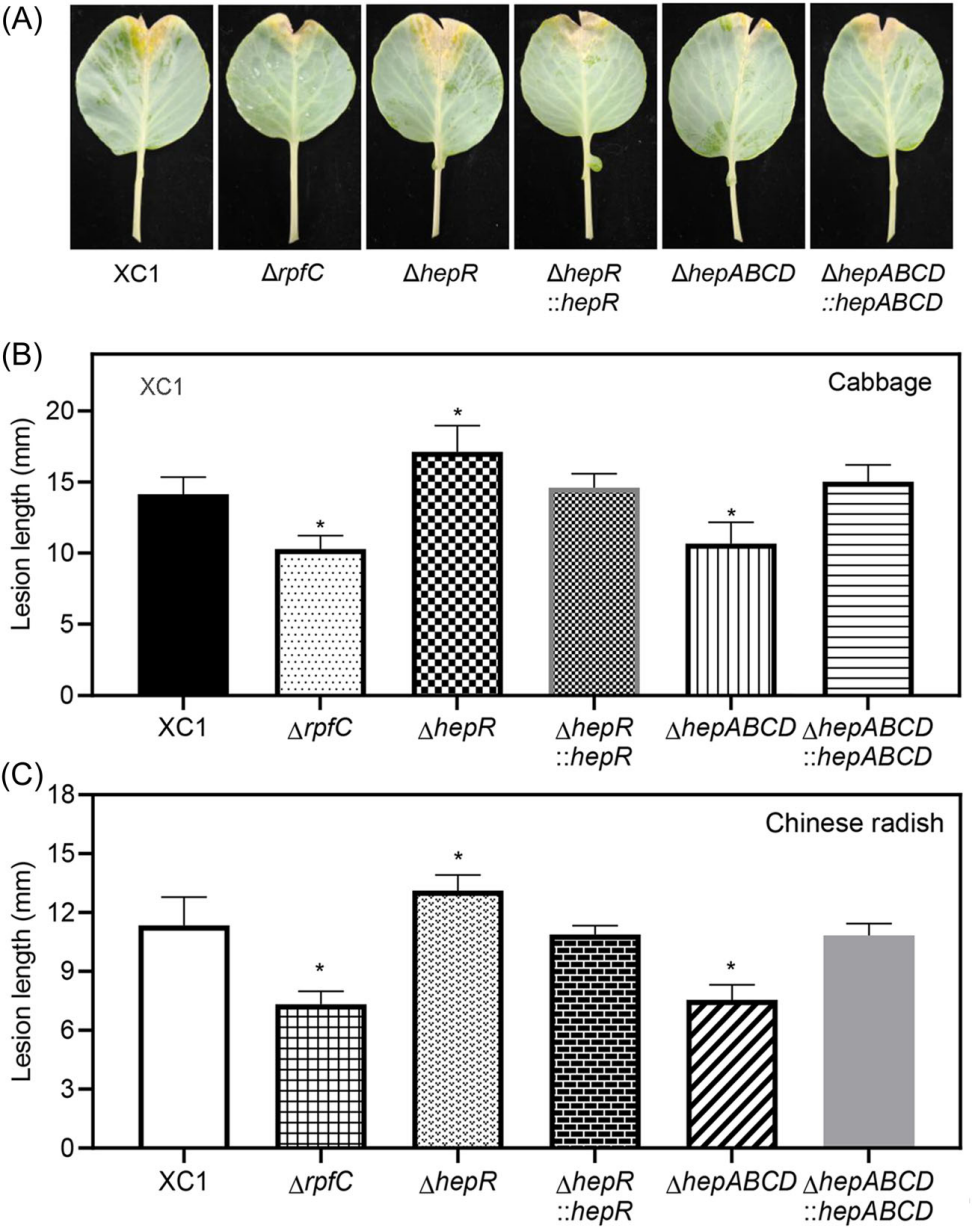
*hepRABCD* is involved in Xcc virulence. (A) Lesions of cabbage leaves infected with XC1, Δ*rpfC*, Δ*hepR*, Δ*hepR::hepR*, Δ*hepABCD*, and Δ*hepABCD::hepABCD* at 15 dpi. (B, C) Quantitative analysis of the lesion length at 15 dpi in cabbage (B) and Chinese radish (C) leaves infected with XC1, Δ*rpfC*, Δ*hepR*, Δ*hepR::hepR*, Δ*hepABCD*, and Δ*hepABCD::hepABCD*, respectively. A total of 15 leaves from 15 plants were inoculated with each strain, and the average lesion lengths with the standard deviation (SD) are shown. The mutant strain Δ*rpfC* was used as a negative control. Statistically significant differences are indicated by one asterisk (*p* < 0.05).

The virulence levels of strains XC1, Δ*hepR*, and Δ*hepABCD* in cabbage and Chinese radish were determined using the leaf‐clipping method (Figure 10). The mutant strain Δ*rpfC* was used as a negative control. At 15 dpi, the average lesion lengths of the strain Δ*hepABCD* in cabbage and Chinese radish were 10.6 and 7.5 mm, respectively (Figure 10B,C), which were significantly shorter than 14.1 mm in cabbage, 11.4 mm in Chinese radish, respectively, infected with the wild‐type strain XC1 (Figure 10). The *hepABCD* complementation strain Δ*hepABCD*::*hepABCD* displayed similar lesion length to the wild‐type XC1 in both cabbage and Chinese radish (Figure 10).

At 15 dpi, the average lesion lengths of strain Δ*hepR* in cabbage and Chinese radish were 17.1 and 13.1 mm, respectively, which were significantly longer than the wild‐type strain XC1 (Figure 10B,C). The *hepR* complementation strain Δ*hepR*::*hepR* displayed similar lesion length to the wild‐type XC1 in both cabbage and Chinese radish (Figure 10B,C).

### The *hepRABCD* cluster is conserved in the phytopathogens *Xanthomonas* and other bacterial strains

A BLAST analysis of the NCBI Nonredundant (Nr) database revealed that the *hepRABCD* cluster was present in the genomes of almost all *Xanthomonas* species (Figure S5). In addition, this gene cluster was also present in *Stenotrophomonas maltophilia* K279a, *Stenotrophomonas pavanii*, *Luteibacter pinisoli* MAH‐14, *Acidovorax avenae* ATCC 19860, *Pseudomonas putida* KT2440, *Pseudomonas protegens* Pf‐5, *Pseudomonas syringae* pv. *syringae* B728a, *Pandoraea oxalativorans* DSM 23570, *Burkholderia glumae* BGR1, and *Burkholderia multivorans* ATCC 17616 (Figure S5). Phylogenetic tree analysis demonstrated that the *hepRABCD* operon in Xcc is evolutionarily close to that in *Xanthomonas campestris* pv. raphani 756C (Figure S6). These findings suggest that sensing and efflux of SA are conserved mechanisms in the phytopathogen *Xanthomonas* and some other bacterial species.

## DISCUSSION

SA is an essential plant defense hormone. Infection by phytopathogens induces SA accumulation, which functions in the amplification of defense gene expression and the production of pathogenesis‐related proteins and toxic antimicrobial compounds. These compounds protect plants from infection^26^. In contrast, successful phytopathogens utilize a variety of mechanisms to protect themselves from toxic compounds produced by host plants. Previous findings have demonstrated that SA produced by cabbage can directly act on the invading Xcc to induce QS signal, DSF turnover^11^. The present study further identified a novel SA sensor HepR and an SA efflux pump system HepABCD in Xcc. We showed that Xcc sensed the SA signal via HepR and then increased the expression of *hepABCD* to activate the efflux of SA from Xcc cells. Following SA sensing, the culture pH increased, and QS signal DSF turnover was induced in Xcc. The expression of the *hepRABCD* gene cluster was upregulated in plant tissue and required for Xcc virulence in Chinese cabbage. These findings reveal a new strategy to ensure phytopathogen survival and virulence in susceptible host plants.

SA perception and signaling in plants have become increasingly understood in the past three decades^5^, ^6^. In addition to the SA receptors NPR1 and NPR3/NPR4, many plant SA‐binding proteins (SABPs) with varied affinities to SA have been identified^27^. This makes SA the “sixth” phytohormone that plays a crucial role in plant growth, development, and defense. Moreover, SA sensing and perception have also been reported in the microbial world. The transcription factor SlyA has been well characterized to upregulate virulence genes in *Salmonella enterica* serovar *Typhimurium* ^28^. The addition of 25 mM SA led to the complete dissociation of SlyA from the target DNA, suggesting that SA could be an effector of SlyA^23^. SA‐SlyA co‐crystal structure analysis demonstrated that SA can bind SlyA and stabilize the SlyA dimmer conformation unfavorable for interacting with the target promoter DNA region^24^. SlyA usually senses SA in a weak affinity. In addition, a LysR‐family transcriptional regulator, CeoR, has been shown to be a putative SA sensor regulating efflux pump genes in *Burkholderia cenocepacia* (genomovar III)^29^. Recently, SA has been shown to bind directly to *Pectobacterium* N‐acyl‐homoserine lactone synthase ExpI, interfering with QS^30^. These findings suggest that SA perception in microbes is mediated by multiple sensors. The present study demonstrated that HepR is an SA sensor in Xcc and is conserved in a wide range of bacterial plant pathogens (Figures 6 and S5). HepR shares a very limited amino acid identity or domain organization with the abovementioned SA sensors in bacterial pathogens (Figure S3). Therefore, HepR likely represents a putative new SA sensor in plant pathogens. Considering the relatively high *K*_d_ (19.3 ± 3.7 μM) in the binding assay of SA and HepR and the unpublished results showing that 4‐hydroxybenzoic acid (4‐HBA) could also be sensed by HepR (Data not shown), HepR is not likely to be a receptor‐specific for SA perception and signaling in Xcc. Further characterization of these SA‐sensed compounds and their signaling pathways in Xcc is needed to fully understand the roles of HepR.

To establish successful infection, plant pathogens have to eliminate the effects of antimicrobial compounds. RND family efflux pumps are recognized as a major means by which bacteria extrude antimicrobials outside the cell. The most relevant roles of the RND family of efflux pumps identified thus far include bacterial virulence, plant–bacteria interactions, transport of QS molecules, and extrusion of various types of toxic compounds^31^, ^32^, ^33^. Their loss or impairment compromises the ability of the pathogenic bacteria *Erwinia*, *Agrobacterium tumefaciens*, *Rhizobium*, and *Pseudomonas* to cause disease in their hosts^26^, ^33^, ^34^. Although SA has been shown to induce the expression of several efflux pump genes, the biological compounds effluxed by these pumps and the biological significance of SA‐dependent efflux pump activation remain elusive in these bacteria. In this study, *hepR* was co‐transcribed with *hepABCD*, which encodes a typical RND family efflux pump. *hepRABCD* expression was induced by SA, and SA bound to HepR to activate the SA efflux pump. Because SA is present at an Xcc infection site, our observation of efflux pump gene induction by SA is interesting and relevant. To the best of our knowledge, this is the first report showing that the phytopathogen Xcc can sense SA and efflux SA via an RND family efflux pump system to promote virulence.

**Figure 11:**
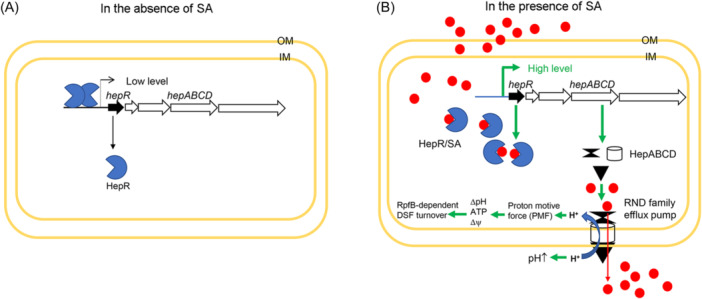
A proposed working model for the SA‐dependent activation of the RND family efflux pump HepABCD. (A) In the absence of SA, the *hepRABCD* cluster is transcribed at a low basal level. The HepR dimer binds to its own promoter P_hep_, which limits *hepRABCD* transcription. (B) In the presence of SA, due to its good membrane‐forming property, SA can easily enter the Xcc cell. Inside the Xcc cell, SA serves as a ligand for the sensor HepR. The HepR/SA complex releases HepR from the promoter P_hep_, which increases *hepRABCD* transcription and results in a high level of HepR, HepA, HepB, HepC, and HepD. The latter four proteins form an RND family efflux pump to pump SA out of the Xcc cell. The activity of the HepABCD RND‐family efflux pump is coupled to the proton gradient across the cell membrane, which likely results in the culture pH increase and induces RpfB‐dependent DSF turnover. Δψ, electrical potential; ΔpH, chemical potential.

Based on previous findings and the present results, we proposed a schematic model for SA sensing and efflux and the roles of the RND family efflux pump in culture pH and DSF turnover in Xcc. In the absence of SA, the *hepRABCD* cluster is transcribed at a low basal level, and the HepR dimer binds its own promoter P_hep_, which limits *hepRABCD* transcription (Figure 11A). Due to its good membrane‐forming properties, SA can easily enter the Xcc cell. Inside the Xcc cell, SA serves as a ligand for the sensor HepR. The HepR/SA complex releases HepR from the promoter P_hep_, which increases *hepRABCD* transcription and results in high levels of HepR, HepA, HepB, HepC, and HepD. The latter four proteins form an RND family efflux pump to pump SA out of the Xcc cell. The activity of the HepABCD RND family efflux pump is coupled to the proton gradient across the cell membrane to form a proton motive force (PMF). PMF further drives the pump efflux to generate a chemical potential (ΔpH) and an electrical potential (Δψ) across the cell membrane, resulting in ATP biosynthesis inside the bacterial cell^35^. Results of our previous study have shown that the increased cytoplasmic pH increases the enzymatic activity of RpfB, inducing DSF turnover^11^. Whether the electrical potential and ATP biosynthesis are also involved in the induction of RpfB‐dependent DSF turnover remains to be elucidated in the future (Figure 11B).

In summary, our data suggest that successful plant bacterial pathogens, such as *Xanthomonas*, can sense and efflux the plant signal SA to enhance tolerance to toxic plant antimicrobial chemicals. The ability of Xcc to co‐opt the plant defense signal SA to activate the multidrug efflux pump may have evolved to ensure Xcc survival and virulence in susceptible host plants. Studies on the specific interactions between SA and HepR, the HepR regulon, and the HepR signaling pathway in Xcc are currently being investigated. These studies may help us to fully understand the complex sensing and signaling that occurs between pathogens and hosts.

## MATERIALS AND METHODS

### Bacterial strains and growth conditions

All bacterial strains and plasmids used are listed in Table S1. The Xcc wild‐type strain XC1 and its derivative strains were cultured in a defined XYS medium (0.7 g l^−1^ K_2_HPO_4_, 0.2 g l^−1^ KH_2_PO_4_, 0.01 g l^−1^ FeSO_4_·7H_2_O, 0.001 g l^−1^ MnCl_2_·4H_2_O, 0.1 g l^−1^ MgCl_2_·6H_2_O, 1 g l^−1^ (NH_4_)_2_SO_4_, 5 g l^−1^ sucrose, and 0.625 g l^−1^ yeast extract, pH 7.0) at 28°C in a laboratory shaker (200 rpm, ZQZY‐75CN). *E. coli* strains were grown in Luria‐Bertani (LB) medium (10 g l^−1^ tryptone, 5 g l^−1^ NaCl and 5 g l^−1^ yeast extract, pH 7.0) at 37°C. When needed, the following antibiotics were added to the medium for selection: rifamycin (Rif, 25 μg ml^−1^), kanamycin (Km, 50 μg ml^−1^), and gentamicin (Gm, 20 μg ml^−1^). Agar was added to the XYS liquid medium at a final concentration of 8 g l^−1^ to prepare an XYS agar plate.

### Gene deletion and complementation

The Xcc deletion mutant was generated following the *sacB*‐mediated double homologous recombination method^18^. Briefly, the upstream and downstream fragments of the target gene were first amplified by PCR using the primers listed in Table S1. The upstream and downstream fragments were cloned into vector pK18mobsacB to obtain a recombinant plasmid using a ClonExpress MultiS One‐Step Cloning Kit (Vazyme). The recombinant plasmid was transformed into *E*. *coli* S17‐1λpir and then transferred to Xcc by di‐parental mating. In succession, colonies with Km and Rif resistance and sucrose sensitivity were selected on NRK (NYG with Km and Rif) and NAS media (NA containing 5% sucrose), respectively. Deletion of the target gene was verified by PCR, and subsequent sequencing was performed using the primers listed in Table S1.

### RNA‐Seq and transcriptome analyses

The Xcc wild‐type strain XC1 was cultured in 50 ml of liquid XYS medium supplemented with 0 or 100 μM SA for 24 h at 28°C. Three biological replicates were prepared. Bacterial cells were collected and the total RNA was extracted. RNA sequencing (RNA‐Seq) was performed by Shanghai Personal Biotechnology Co., Ltd. using the Illumina HiSeq system. Principal component analysis (PCA) based on gene expression was performed on each sample using the DESeq package in R (www.r-projec.org). The correlation of gene expression levels between samples was expressed by Pearson's correlation coefficient. DESeq was used for the differential analysis of gene expression, and differentially expressed genes were screened for expression difference ploidy based on |log_2_(FoldChange)|>1 and *p* < 0.05. Volcano plots of differentially expressed genes were created using the ggplots2 package in R.

### Protein expression and purification

The HepR open reading frame (ORF) was amplified by PCR and cloned into the expression vector pET28a, and the recombinant plasmid was transformed into *E*. *coli* BL21(DE3). For protein expression, a single colony was inoculated into LB with Km and cultivated for 8 h at 37°C. The starting culture was then transferred to 500 ml liquid LB with Kan and grown at 37°C to OD_600_ = 0.6. Bacteria were then induced with 0.1 mM isopropyl‐β‐d‐thiogalactopyranoside (IPTG) and cultivated for 16 h at 16°C. Bacterial cells were collected by centrifugation at 4°C and 4000*g* for 10 min and washed twice using 1× phosphate‐buffered saline (PBS). Bacterial cells were resuspended using HEPES buffer A (25 mM HEPES 150 mM NaCl, 10 mM imidazole, pH 7.4) and disrupted by sonication. The supernatant was collected after centrifugation at 13,800*g* for 40 min at 4°C and loaded onto the Ni‐NTA column (Smart‐Lifesciences). The column was further washed with 50 ml buffer B (25 mM HEPES 150 mM NaCl, 25 mM imidazole, pH 7.4) and was eluted with 250 mM imidazole HEPES buffer. The eluted fractions were pooled and tested by sodium dodecyl sulfate‐polyacrylamide gel electrophoresis (SDS‐PAGE). The fraction containing His‐tagged HepR was concentrated and loaded into a Superdex 75 gel filtration column (GE Healthcare) equilibrated with a nonimidazole buffer (25 mM HEPES and 150 mM NaCl, pH 7.4).

### EMSA

EMSA was performed using the established protocol^25^. Briefly, different concentrations of purified His‐HepR were mixed with Cy5‐labeled promoter fragment P_hep_ in 20 μl of EMSA buffer (20 mM Tris pH 7.9, 2 mM DTT, 10 mM MgCl_2_, 5% glycerol, 40 μg BSA, 100 ng sperm DNA) and then loaded on a 4.5% nondenaturing polyacrylamide gel for electrophoresis in 0.5× TBE buffer for 90 min. The Cy5 fluorophore in the gel was detected using an Amersham Typhoon RGB Biomolecular Imager (Cytiva).

### Deoxyribonuclease I (DNase I) protection footprint assay

For the preparation of fluorescent FAM‐labeled probes, the promoter fragment P_hep_ was amplified at 25 cycles by PCR using the primers T7(FAM) and M13R and the template plasmid pUCm‐T‐P_hep_. DNase I footprint assays were performed at Shanghai Biotechnology Corporation using previously described protocols^36^. Four‐hundred nanograms of the FAM‐labeled probe was incubated with HepR for 30 min at 25°C. Then, 0.015 units of DNase I and 100 nmol CaCl_2_ were added to the reaction and incubated for a further 1 min at 37°C. The reaction was stopped by adding 140 µl of DNase I termination solution (200 mM sodium acetate, 30 mM EDTA, and 0.15% SDS). The remaining DNA was extracted for sequencing.

### ITC and SPR assays

The binding affinity of SA and HepR was measured using ITC and SPR following the protocols provided by the manufacturers. Both the protein HepR and SA were prepared in HEPES buffer (25 mM HEPES 150 mM NaCl, pH 7.4) before titration, and all solutions for titration were centrifuged at 11,200*g* for 10 min. For each ITC measurement, 400 μl of HepR protein was injected into the cuvette, and the excess protein sample was aspirated using a syringe. The SA molecule was then aspirated using a burette, which should be free of air bubbles. The titration temperature was set at 18°C, and the stirring speed was set at 750 rpm. The HepR and SA binding capacity was measured in a MicroCal ITC200 (GE Healthcare).

The SPR assay for HepR–SA interaction analysis was performed using a Biacore 8K (Cytiva) with running PBS supplemented with 0.25% Tween at 25°C. His‐tagged HepR was immobilized on Sensor Chip CM5 using a standard amine‐coupling procedure in 10 mM sodium acetate (pH 5.0). SA was serially diluted and injected onto a sensor chip at a flow rate of 30 μl/min for 120 s, followed by 120 s of buffer flow. The *K*_d_ value was derived using the enclosed evaluation software.

### Cytoplasmic SA extraction and detection

To extract the cytoplasmic SA in Xcc, 50 ml of cultures were collected at 12, 24, and 36 h and centrifuged at 10,000 rpm for 30 min at 4°C, and the bacterial cell pellets were washed three times with 1× PBS. Subsequently, the bacteria were suspended in 10 ml of B‐PER® Bacterial Protein Extraction Reagent and reacted for 30 min at room temperature to fully lyse the cells. After centrifugation at 12,000 rpm for 10 min, the supernatant was collected, and SA was extracted as previously described by Zhou et al.^37^ Briefly, 10 ml of the collected supernatant was pH adjusted to 4.0 and extracted with 20 ml of ethyl acetate. The ethyl acetate fraction was then collected and evaporated using a rotary evaporator. The remaining residues were dissolved in 0.1 ml of methanol.

The quantitative analysis of cytoplasmic SA levels was performed using high‐performance liquid chromatography (HPLC) combined with triple quadrupole tandem mass spectrometry (HPLC‐QqQ‐MS/MS) (Agilent)^25^. Briefly, 10 μl of extract was loaded onto a Zorbax Eclipse XDB‐C18 column (4.6 × 150 mm, 5 μm; Agilent) for substance chromatographic separation. The column was then eluted with methanol containing 0.1% formic acid and water at a flow rate of 0.4 ml min^−1^ for 40 min at a volume ratio of 40/60. Quantitative analysis was performed using an electrospray ionization (ESI) ion source triple quadrupole tandem mass spectrometer (Agilent). The MS spectra were recorded using multiple reaction monitoring (MRM) mode. The cytoplasmic SA level was expressed as the SA concentration per 1 × 10^9^ bacteria.

### GUS activity assay

The construction of *gusA*‐based reporter strains and quantitative GUS activity assays were performed as previously described^38^. Briefly, the reporter strains were grown in XYS medium at 28°C. Bacterial cells were collected, centrifuged at 13,800*g* for 10 min, and washed once with PBS buffer (1×, pH 7.4). The bacterial cells were then suspended in 1 ml PBS buffer and disrupted with the addition of 20 μl of 0.1% (w/v) SDS solution and 40 μl of chloroform. The GUS activities were measured by detecting the fluorescence intensity based on an excitation wavelength of 365 nm and an emission wavelength of 455 nm, using 4‐methylumbelliferone‐d‐glucuronide as the substrate.

### Determining the transcriptional start site of the *hep* gene cluster by rapid amplification of cDNA ends (RACE)

We used a 5′ RACE kit (Sangon) to determine the transcriptional start site of the *hepRABCD* cluster. Briefly, XC1 was used to inoculate XYS medium for 12 h at 28°C, and total RNA was extracted using an RNA extraction kit (Vazyme). The cDNA fragments were amplified using the primers provided in the kit, and a poly(C) tail sequence was added to the 3′ end of the cDNA using terminal deoxynucleotide transferase. The tailed cDNA was then amplified using the RACE adapter primer and the outer primer (5′ adaptor primer: 5′‐GCTGTGAACGATACGCTACGTAACGGCATGACAGTGGGGGGGGGGGGG‐3′; 5′ outer primer: 5′‐GCTGTCAACGATACGCTACGTAAC‐3′). The PCR products were then cloned into pUCm‐T for sequencing.

### Bioinformatics analysis

All DNA sequences, amino acid sequences, and genome sequences were downloaded from NCBI (https://www.ncbi.nlm.nih.gov/). Protein domain organization was predicted using the SMART program (https://smart.embl.de/smart/set_mode.cgi?NORMAL=1). Promoter prediction was performed using the website Prokaryote Promoter Prediction (http://genome2d.molgenrug.nl/g2d_pepper_promoters.php). Multiple sequence alignment analysis was conducted using Clustal Omega (https://www.ebi.ac.uk/Tools/msa/clustalo/).

### Bacterial pathogenicity assay

Pathogenicity assays were performed as previously described^25^. Briefly, the bacterial suspensions used in the pathogenicity assays were adjusted to 1 × 10^8^ colony‐forming units (CFUs)/ml. Two‐month‐old cabbage plants (Jingfeng‐1) and Chinese radish (Manshenghong) were inoculated by making leaf incisions. A total of 15 leaves from 15 plants were inoculated with each strain. After inoculation, the infected plants were covered with the transparent plastic film and kept in a growth chamber set at 28°C with 80% relative humidity. At 14 days postinoculation, the lesion length was measured.

### Statistical analyses

A one‐way analysis of variance was used for multiple comparisons. The statistical test values represent statistical significance, followed by the least significant difference test. Data are presented as means ± standard deviation (SD). All experiments were performed at least three times independently.

## AUTHOR CONTRIBUTIONS

**Kai Song**: Conceptualization (equal); data curation (lead); formal analysis (equal); investigation (equal); methodology (equal); project administration (equal); writing—review and editing (equal). **Ruifang Li**: Data curation (equal); methodology (equal). **Ying Cui**: Data curation (equal); formal analysis (equal); methodology (equal). **Bo Chen**: Formal analysis (equal); methodology (equal). **Lian Zhou**: Formal analysis (equal); methodology (equal). **Wenying Han**: Data curation (equal); formal analysis (supporting). **Bo‐Le Jiang**: Conceptualization (equal); data curation (equal); writing—review and editing (equal). **Ya‐Wen He**: Data curation (equal); funding acquisition (lead); project administration (equal); supervision (lead); writing—original draft (lead); writing—review and editing (lead).

## ETHICS STATEMENT

This study did not involve human subjects and animals.

## CONFLICT OF INTERESTS

The authors declare no conflict of interests.

## Supporting information

Supporting information.

## Data Availability

The authors confirm that the data supporting the findings of this study are available within the article and its supporting information materials.
