# Supplementary material for: The phytopathogen Xanthomonas campestris senses and effluxes salicylic acid via a sensor HepR and an RND family efflux pump to promote virulence in host plants
Source: mLife. 2024 Sep 16;3(3):430–44. doi: 10.1002/mlf2.12140 (PMC11442134; doi:10.1002/mlf2.12140)
Supplement: Supplementary file 1 — Supporting information. [file MLF2-3-430-s001.pdf]

RNA-Seq analysis pipline

XC1 grown in XYS medium in the absence and presence of 100 μM SA for 24 hours

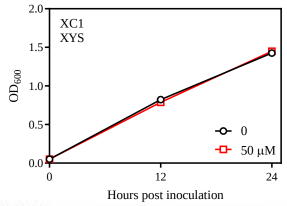

↓

Total RNAs extraction, purification and quality analysis

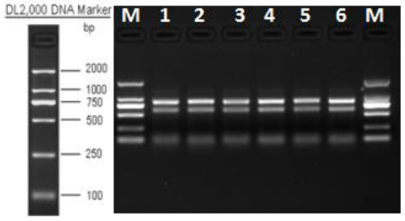

↓

Sequencing

↓

Principal component analysis of differentially expressed genes

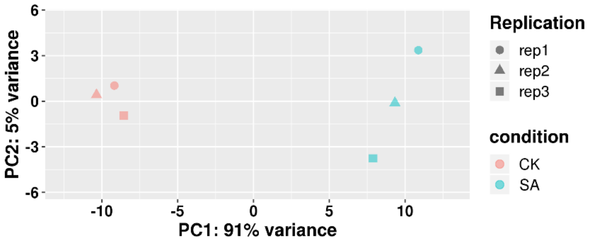

↓

Correlation analysis of gene expression pattern in three groups

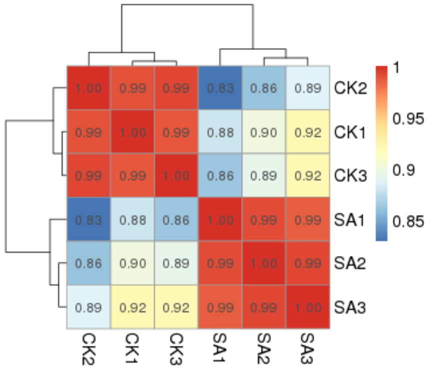

↓

Volcano picture of differentially expressed genes (change > 2.0-fold)

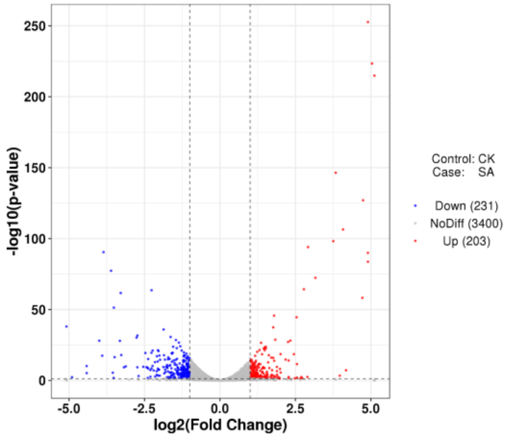

↓

A table to summarize how many genes upregulated(>2 fold) and how many gene downregulated(<0.5)

↓

SA-regulated genes

Figure S1. RNA-Seq analysis of the SA-treated XC1 strain and SA-regulated genes in XC1.

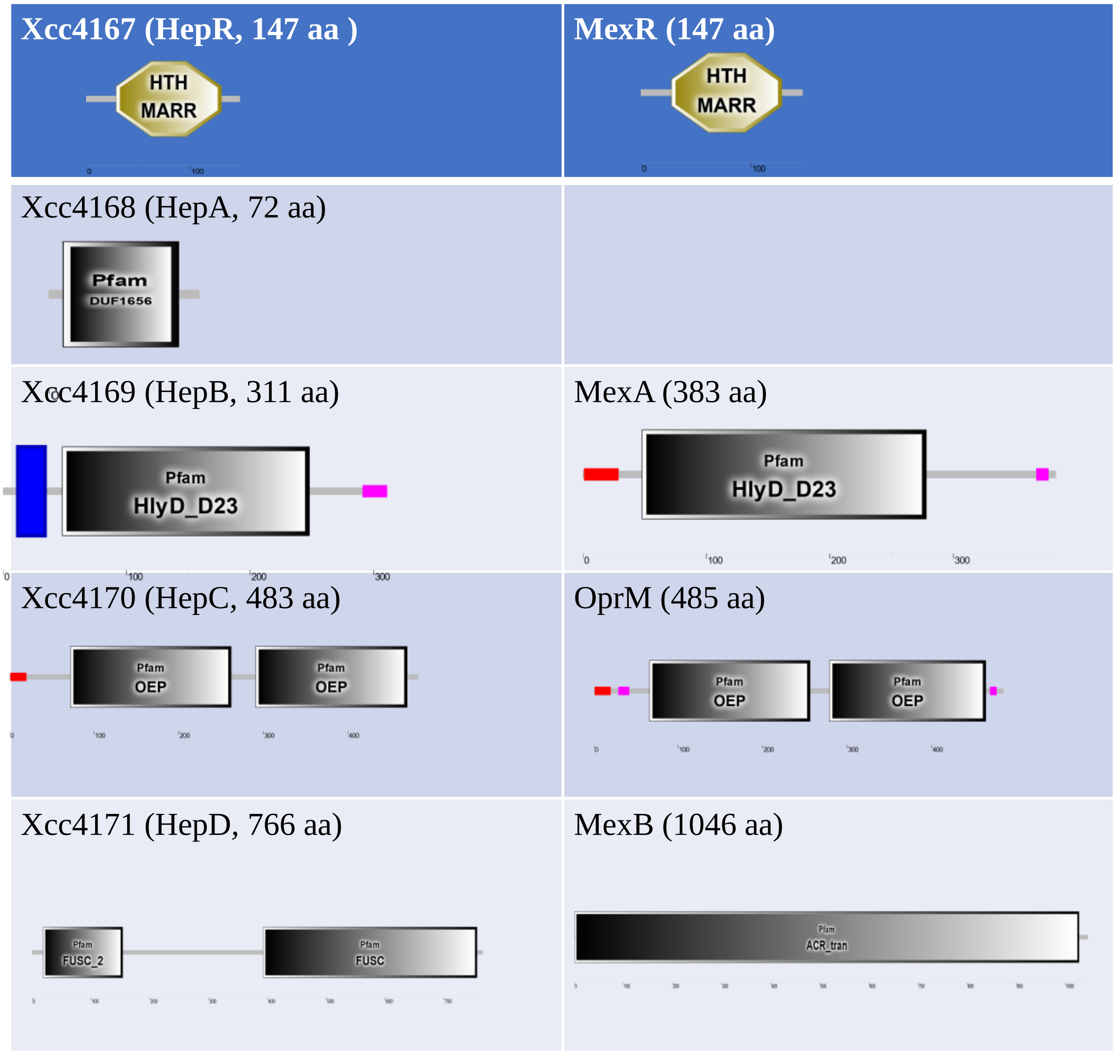

Figure S2. Domain analysis of the RND-family efflux pumps HepRABCD in *Xanthomonas campestris* pv. *campestris* and MexR-MexA-OprM-MexB in *Pseudomonas aeruginosa* PAO1.

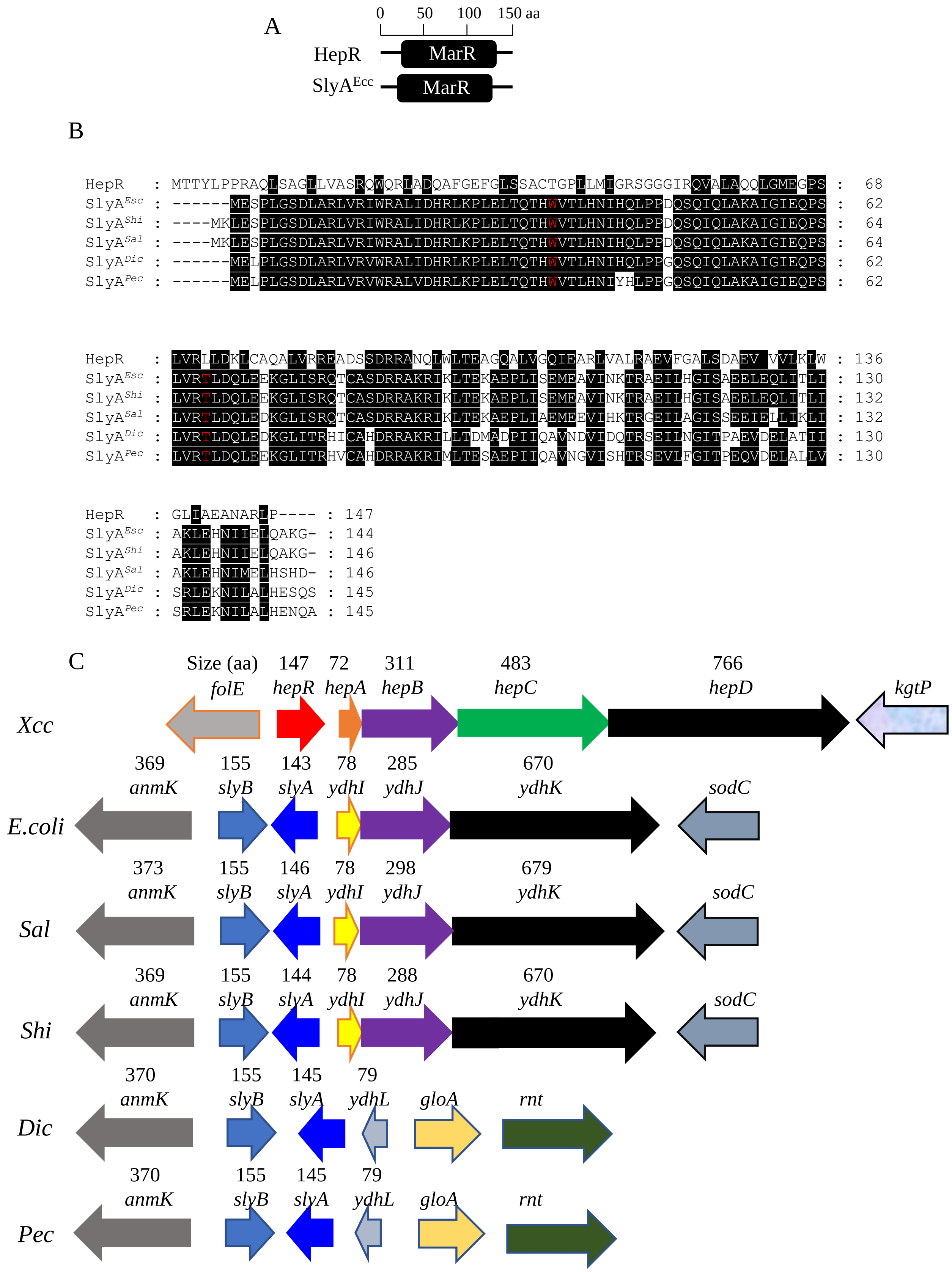

Figure S3. HepR is a novel SA sensor. (A) Domain analysis of HepR and SlyA^{ECC}. (B) Multiple sequence alignment analysis of HepR and SlyA homologs. (C) The *hepR* flanking genes in Xcc and *slyA* flanking genes in the strains of *E. coli*, *Salmonella* (Sal), *Shigella* (Shi),, *Dickeya* (Dic), and *Pectobacterium* (Pec).

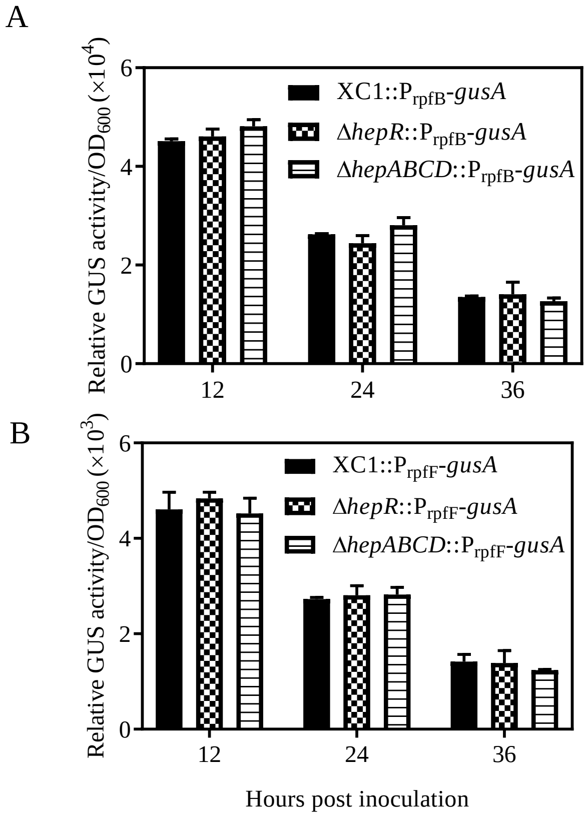

Fig. S4. HepR does not directly regulate the expression of *rpfF* and *rpfB* . (A) GUS activity of reporter strain XC1::P_{rpfB}-*gusA*, Δ*hepR*::P_{rpfB}-*gusA* and Δ*hepABCD*::P_{rpfB}-*gusA* in XYS medium. (B) GUS activity of reporter strain XC1::P_{rpfF}-*gusA*, Δ*hepR*::P_{rpfF}-*gusA* and Δ*hepABCD*::P_{rpfF}-*gusA* and in XYS medium.

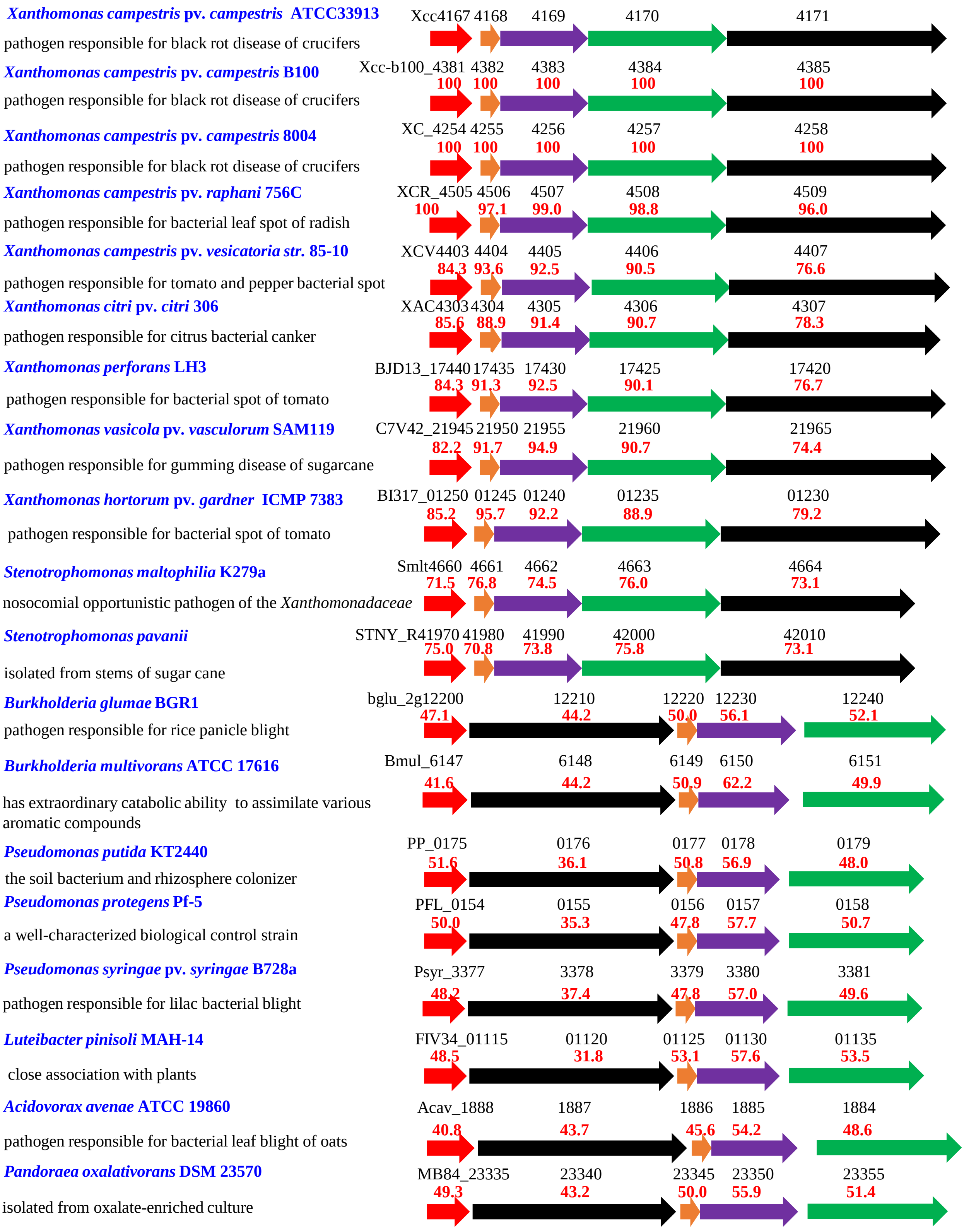

Figure S5. The gene cluster *hepRABCD* is conserved in a range of bacterial species.

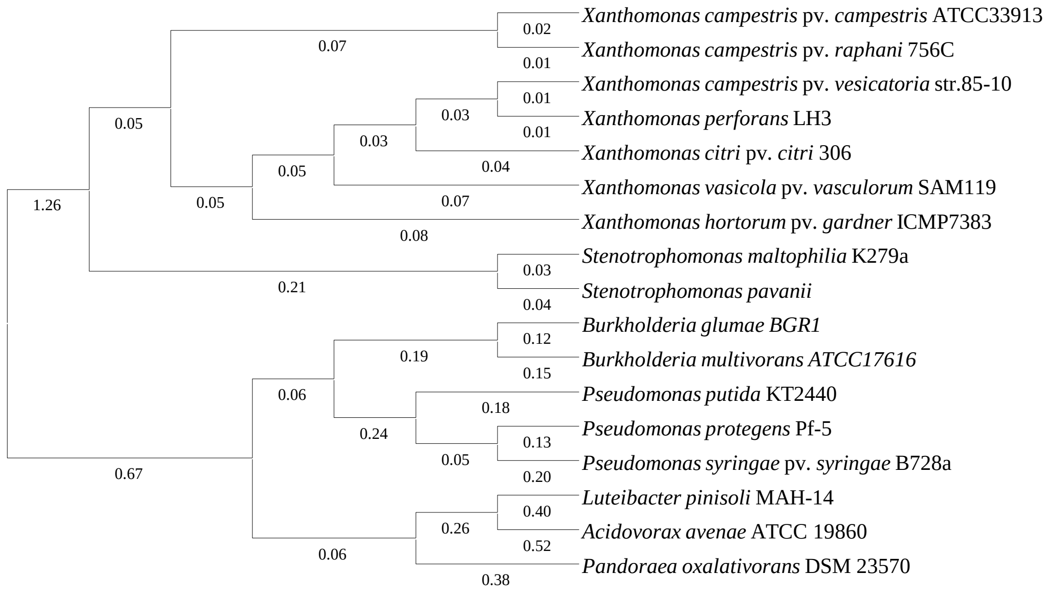

Figure S6. Phylogenetic tree analysis of HepABCD and RND transport systems in different bacteria using the maximum likelihood method.

Table S1. Bacterial strains used in this study

Strain	Properties / characteristics	Reference / source
***Xcc* Strains**		
XC1	*Xcc* wild-type strain, Rif^R	Lab stock
Δ*hepR*	The *hepR* in-frame deletion mutant, Rif^R	This study
Δ*hepR*::*hepR*	The *hepR* in-frame deletion mutant complemented with a single copy of *hepR* inserted at the *att*Tn*7* site on its chromosome, Rif^R	This study
Δ*hepABCD*	The *hepABCD* in-frame deletion mutant, Rif^R	This study
Δ*hepABCD*::*hepABCD*	The *hepABCD* in-frame deletion mutant complemented with a single copy of *hepABCD* inserted at the *att*Tn*7* site on its chromosome, Rif^R	This study
Δ*hepRABCD*	The *hepR hepABCD* double deletion mutant, Rif^R	This study
XC1::P_{hep}-*gusA*	XC1 harboring a single copy of T0T1-P_{hep}-*gusA* at the attTn7 site on its chromosome, Rif^R	This study
XC1::P_{hepA}-*gusA*	XC1 harboring a single copy of T0T1-P_{hepA}-*gusA* at the attTn7 site on its chromosome, Rif^R	This study
Δ*hepR*::P_{hep}-*gusA*	The Δ*hepR* harboring a single copy of T0T1-P_{hep}-*gusA* at the attTn7 site on its chromosome, Rif^R	This study
XC1::$P_{hep\text{-}GCT\text{-}}$*gusA*	XC1 harboring a single copy of T0T1-$P_{hep\text{-}GCT}$-*gusA* at the attTn7 site on its chromosome, Rif^R	This study
XC1::P_{pobA}-*gusA*	XC1 harboring a single copy of T0T1-P_{pobA}-*gusA* at the attTn7 site on its chromosome, Rif^R	[Chen *et al*. 2020]
XC1::P_{hep}-*gusA*(pBBR)	The XC1::P_{hep}-*gusA* harboring the expression plasmid pBBR1MCS-2, Rif^R Kan^R	This study
Δ*hepR*::P_{hep}-*gusA*(pBBR)	The Δ*hepR*::P_{hep}-*gusA* harboring the expression plasmid pBBR1MCS-2, Rif^R Kan^R	This study
Δ*hepR*(*hepR*)::P_{hep}-*gusA*	The Δ*hepR*::P_{hep}-*gusA* harboring the expression plasmid pBBR-*hepR*, Rif^R Kan^R	This study
XC1 (*pchAB)*	XC1 harboring the expression plasmid pBBR-*pchAB*, Rif^R Kan^R	This study
Δ*hepR* (*pchAB*)	The *hepR* in-frame deletion mutant harboring the expression plasmid pBBR-*pchAB*, Rif^R Kan^R	This study
Δ*hepABCD* (*pchAB*)	The *hepABCD* in-frame deletion mutant harboring the expression plasmid pBBR-*pchAB*, Rif^R Kan^R	This study
***E. coli* strains**		
DH5α	*E. coli* F– Φ80lacZΔM15 Δ(lacZYA-argF) U169 recA1 endA1 hsdR17 (rK–, mK+) phoA supE44 λ– thi-1 gyrA96 relA1	Lab stock
S17-1	res^- pro mod^+ integrated copy of RP4, mob^+	Lab stock
BL21(DE3)	*E. coli* B F- dcm ompT hsdS(r_B- m_B-) gal $[malB^+]_{K\text{-}12}(\lambda^S)$	Lab stock
pRK2013	Triparental mating helper strain, Kan^R	Lab stock

Table S2. Plasmids used in this study

Plasmids	Properties / characteristics	Reference / source
pK18mobsacB	A mobilizable vector, allows for selection of double crossover in *Xcc*, Kan^R	[Schäfer *et al.*, 1994]
pK18-*hepR*	*Xcc hepR* deletion cassette in pK18mobscaB, Kan^R	This study
pK18-*hepABCD*	*Xcc hepABCD* deletion cassette in pK18mobscaB, Kan^R	This study
pK18-*hepRABCD*	*Xcc hepRABCD* deletion cassette in pK18mobscaB, Kan^R	This study
pBBR1MCS-2	Plasmid for gene complementation, Kan^R	[Kovach *et al.*, 1995]
pBBR-*pchAB*	*Pseudomonas aeruginosa pchAB* cloned in pBBR1MCS-2, Kan^R	This study
pBBR-*hepR*	*hepR* cloned in pBBR1MCS-2, Kan^R	This study
pBBR-*hepABCD*	*hepABCD* cloned in pBBR1MCS-2, Kan^R	This study
pET28a	His-tag protein expression vector, Kan^R	NEB
pET28a-*hepR*	pET28a containing the coding sequence of *hepR*, Kan^R	This study
pMD18T-T0T1-*gusA*	Promoter probe cassette T0T1-MCS-*gusA* cloned in pMD18-T simple, Amp^R	[Zhao *et al.*, 2014]
mini-Tn7T-Gm	A versatile mini-Tn7 delivery vector mini-Tn7T-Gm, Gm^R	[Choi and Schweizer, 2006]
mini-Tn7-T0T1-*gusA*	mini-Tn7T-Gm containing a T0T1 terminator fused to the coding region for *gusA*, Gm^R	This study
mini-Tn7-T0T1-P_{hep}-*gusA*	mini-Tn7T-Gm containing a T0T1 terminator and 531 bp promoter region of *hepR* fused to the coding region for *gusA*, Gm^R	This study
mini-Tn7-T0T1-P_{hepA}-*gusA*	mini-Tn7T-Gm containing a T0T1 terminator and 437 bp promoter region of *hepA* fused to the coding region for *gusA*, Gm^R	This study
mini-Tn7-T0T1-$P_{hep\text{-}GCT}$-*gusA*	mini-Tn7T-Gm containing a T0T1 terminator and 531 bp promoter region point mutation ATC:GCT of *hepR* fused to the coding region for *gusA*, Gm^R	This study
pUCmT-P_{hep}	The 291 bp promoter region of *hepR* cloned in pUCmT, Amp^R	This study

References:

Chen B, Li RF, Zhou L, Qiu JH, Song K, Tang JL, He YW. (2020) The phytopathogen *Xanthomonas campestris* utilizes the divergently transcribed *pobA*/*pobR* locus for 4-hydroxybenzoic acid recognition and degradation to promote virulence. *Mol Microbiol*. 114(5):870-886.

Choi K H, Schweizer H P. (2006) Mini-Tn7 insertion in bacteria with single attTn7 sites: example *Pseudomonas aeruginosa*. *Nat Protoc*. 1(1): 153-161.

Kovach M E, Elzer P H, Hill D S, Robertson G T, Farris M A, Roop R M, Peterson K M. (1995) Four new derivatives of the broad-host-range cloning vector pBBR1MCS, carrying different antibiotic-resistance cassettes. *Gene*. 166(1): 175-176.

Schäfer A, Tauch A, Jäger W, Kalinowski J, Thierbach G, Pühler A. (1994) Small mobilizable multi-purpose cloning vectors derived from the *Escherichia coli* plasmids pK18 and pK19: selection of defined deletions in the chromosome of *Corynebacterium glutamicum*. *Gene*. 145(1): 69-73.

Zhao Z H, Xiong L, Shen C W, Sun Q B, Zou L F, Chen G Y. (2014) Construction of two promoter-probe vectors suitable for pathogenicity-related gene expression analysis in *Xanthomonas oryzae* pv. *oryzicola*. *Acta Phytopathol Sin*. 44(05): 504-511.

Table S3. Oligonucleotide primers used in this study

Application	Primers	Sequence (5' to 3')
hepR deletion	*hepR*_F1	ctatgacatgattacgaattcGCTGACGCCGCGCTTGTGG
	*hepR*_R1	CCCGACCAACGCCTGCTGGGCGCGGGGAGGAAGATAG
	*hepR*_F2	CAGGCGTTGGTCGGGCAGATC
	*hepR*_R2	caggtcgactctagaggatccTCCGGCGCCACCTGAACCA
hepABCD deletion	*hepABCD*_F1	ctatgacatgattacgaattcGCTGCCTGCCGGGGGTTTAGT
	*hepABCD*_R1	CCGCGGCCAGTACCAGAGCAGGTAGGCCAGCGTCATCAAC
	*hepABCD*_F2	TGGTACTGGCCGCGGTGTTTTTG
	*hepABCD*_R2	caggtcgactctagaggatccCGGCGCAGCTGGAGGATGTTCA
promoter of *hep* for GUS activity	P_{hep}-F	taggacaaatccgccaagcttACTGGGCGATCTGTGCGGTCAT
	P_{hep}-R	gttagttaggcccggggatccGCCGGCACTGAGCTGGGCGC
promoter of *hepA* for GUS activity	P_{hepA}-F	taggacaaatccgccaagcttTGGCGAGTTCGGCCTGTCCAG
	P_{hepA}-R	gttagttaggcccggggatccCGGCCACATATCCTGGGGAAGGGA
HepR protein expression	HepR_F	GGGAATTCcatatgATGACTACCTATCTTCCTCCCCGCGCC
	HepR_R	CGggatccCTAGGGCAGGCGCGCGTTCG
$P_{hep\text{-}GCT}$ point mutation	ATC:GCT_F	GAATTGATAGCATCCTGCTTATTGTGATGACTA
	ATC:GCT_R	TAGTCATCACAATAAGCAGGATGCTATCAATTC
promoter of *hep* for EMSA	Cy5-P_{hep}-F	agccagtggcgataaggggTAACGGATGAATCGGGCTGGTC
	Cy5-P_{hep}-R	agccagtggcgataagcggTGCAGGCGCTGGACAGG
promoter of *hep* for foot-printing	FP-P_{hep}-F	GGGTAACGGATGAATCGGGCTGGTC
	FP-P_{hep}-R	CGGTGCAGGCGCTGGACAGG
RT-PCR analysis	*hepR-hepA*-F	CAGGCGTTGGTCGGGCAGAT
	hepR-hepA-R	CCAACGCATGAGGGAAAACAGG
	hepA-hepB-F	GACGCTGGCCTACCTGCTCAACA
	hepA-hepB-R	CCACTGCGGCCACCACCAC
	hepB-hepC-F	CACCGCCACCGTCACCATCCTG
	hepB-hepC-R	GCTGACGCCGGCTTCCACTTCATAG
	hepC-hepD-F	GCGCACGCCCTACCTATCCAGC
	hepC-hepD-R	CCAGTACGGGCGCGACAGATTG
qRT-PCR analysis	*hepR*-qRT-F	GTACGGCTGCTGGACAAG
	hepR-qRT-R	TCAACACCACCTCCACCTC
	hepB-qRT-F	GAAGATGCCGAAGTGCGC
	hepB-qRT-F	TCCAGCACTGCCACCACC
	hepC-qRT-F	TCGACTATGAAGTGGAAGCC
	hepC-qRT-F	GTTGAACACGGGCAGTTT
	hepD-qRT-F	ACGGCATATCGACTACGG
	hepD-qRT-F	CCCAGGTAAGGAAGGACAC
Construction of transcriptional activity reporter vectors	T0T1-F	AGAgaattcGACTCCTGTTGATAGATCCAG
	gusA-R	AACggtaccTCATTGTTTGCCTCCCTGCT
